# Scrutinizing the untapped potential of emerging ABSe_3_ (A = Ca, Ba; B = Zr, Hf) chalcogenide perovskites solar cells

**DOI:** 10.1038/s41598-024-80473-4

**Published:** 2025-01-27

**Authors:** Dhineshkumar Srinivasan, Aruna-Devi Rasu Chettiar, Eupsy Navis Vincent Mercy, Latha Marasamy

**Affiliations:** Facultad de Química, Materiales-Energía, Universidad Autónoma de Querétaro, Santiago de Querétaro, C.P.76010 Querétaro México

**Keywords:** Solar cells, Solar cells

## Abstract

ABS_3_chalcogenide perovskites (CPs) are emerging as promising alternatives to lead halide perovskites due to their unique properties. However, their bandgap exceeds the Shockley-Queisser limit. By substituting S with Se, the bandgap is significantly reduced, shifting it from the visible into the near-infrared region. Hence, we have investigated the potential of Se-based absorbers with device structure FTO/TiO_2_/ABSe_3 _(A = Ca, Ba; B = Zr, Hf)/NiO/Au using SCAPS-1D. We analyzed the critical parameters impacting each layer of the solar cell. Notably, we achieved an enhanced light absorption (~ 26.5%) at an optimal absorber thickness (500 nm), intensifying carrier generation. Additionally, we observed an increase in V_OC _(1.03 V) due to improved quasi-Fermi level splitting and a reduction in energy loss (0.45 V) across all solar cells with an optimal absorber carrier concentration (10^16  ^cm^−3^). Overall, the optimization resulted in improvements in PCE by the difference of 20.14%, 20.44%, 14.33%, and 14.56% for CaZrSe_3_, BaZrSe_3_, CaHfSe_3_, and BaHfSe_3 _solar cells, respectively. The maximum PCE of over 30% was attained for both CaZrSe_3_and BaZrSe_3 _solar cells, attributed to their narrow bandgap, enhanced light absorption (53.60%), high J_SC _(29 mA/cm^2^), and elevated generation rate of 1.19 × 10^22 ^cm^−2^s^−1^. Thus, these significant outcomes highlight the potential of these absorbers for fabricating high-efficiency CP solar cells.

The growing global demand for energy underscores the need for sustainable resources, particularly through photovoltaic (PV) technology that converts solar energy into electricity^1^. Lead halide perovskites (α-FAPbI_3_) have achieved notable power conversion efficiency (PCE) of up to 26.1%^2^. However, challenges like toxicity and instability against moisture and heat hinder their commercial use. Researchers are exploring lead-free alternatives like bivalent Sn^2+^ and Ge^2+^, but their rapid oxidation and scarcity limit practicality^3^. Integrating trivalent cations like Bi^3+^ and Sb^3+^ in place of Pb^2+^has been tested, but the PCE remains lower due to unfavorable optoelectronic properties^4^. Ongoing research aims to find materials that combine the advantages of lead halide perovskites while overcoming these challenges. In this context, chalcogenide perovskites have garnered significant interest in the scientific community due to their advantageous properties, which include a non-toxic composition, remarkable structural stability, and highly promising electronic characteristics. In a pivotal study conducted in 2015 by Sun et al., a diverse range of ABS_3_ chalcogenide perovskites absorbers was identified as potential candidates. Notable examples include BaZrS_3_, CaZrS_3_, SrZrS_3_, BaHfS_3_, CaHfS_3_, SrHfS_3_, CaTiS_3_, BaZrSe_3_, CaZrSe_3_, SrZrSe_3_, BaHfSe_3_, SrHfSe_3_ and CaTiSe_3_^5^. Subsequent research efforts, especially by Yanbing Han et al. and Yarun Liang et al., focused on the synthesis of SrHfO_3_ films through the technique of magnetron sputtering, which were later subjected to sulfurization at an elevated temperature of 1000 °C. This process successfully yielded SrHfS_3 _films^6,7^. Similarly, a study by Zhonghai Yu et al. demonstrated the effective synthesis of BaHfS_3_, BaZrS_3_, SrHfS_3_, and SrZrS_3_ using sputtering techniques to produce the perovskite precursors BaHfO_3_, BaZrO_3_, SrHfO_3_, and SrZrO_3 _followed by sulfurization over a temperature range of 650 °C to 1050 °C^8^. Despite the intriguing properties exhibited by BaZrS_3_, SrZrS_3_, BaHfS_3_, and SrHfS_3_, the high synthesis temperatures, typically in the range of 900 °C to 1000 °C, present considerable hurdles for practical solar cell fabrication. This is largely due to the limitations of the substrates and electron transport layers (ETLs), which often cannot tolerate such extreme conditions. Recent advancements in synthesis techniques have made headway in addressing these challenges. For example, Comparotto et al. reported successfully synthesizing BaZrS_3 _thin films via a sputtering-sulfurization approach at notably lower temperatures, specifically below 600 °C^9^. Building on this, Nag et al. synthesized BaZrS_3_ nanocrystals at 600 °C, which were subsequently formulated into an ink for film coatings. Yu et al. further improved the synthesis process by obtaining BaZrS_3 _thin films at an even lower temperature of 500 °C through innovative modifications to the chemical reaction pathway^10^. Additionally, Lorenza Ramagnoli et al. introduced a straightforward synthetic procedure for producing BaZrS_3_ and BaHfS_3 _at temperatures as low as 500 °C, providing another viable pathway for initiating the fabrication of these promising materials^11^. Notably, Yang et al. and Zilevu et al. embarked on developing a colloidal synthesis method for creating BaZrS_3 _nanopowders at temperatures below 350 °C. This underscores the growing potential for solution-based processing techniques in the synthesis of chalcogenide perovskites^12,13^. Most recently, in 2023, Vincent et al. unveiled a groundbreaking solution-based methodology for synthesizing BaZrS_3 _thin films. This innovative process involves heat-treating precursor films in an S-rich environment at moderate temperatures, ranging from 500 °C to 575 °C^14^. This development notably enhances the compatibility of BaZrS_3_ with conventional solar absorber materials, such as Si, CIGS, and CdTe. Despite these significant advancements in synthesis techniques, it is worth mentioning that, to date, only one report has been published regarding the fabrication of BaZrS_3 _solar cells. This report achieved a PCE of merely 0.17%^15^, indicating that while the materials hold promise, further research and development are crucial to unlock their full potential in PV applications.

Recent studies indicate that substituting S with Se in ABS_3_chalcogenide perovskites can significantly reduce the bandgap from the visible spectrum to the near-infrared region^16^. This modification enhances the material’s suitability for PV applications, presenting a promising avenue for solar energy technology. Research has identified four Se-containing absorbers: CaZrSe_3_, BaZrSe_3_, CaHfSe_3_, and BaHfSe_3_, with bandgaps of 1.4 eV, 1.35 eV, 1.65 eV, and 1.5 eV, respectively. These materials exhibit favorable optical and electrical properties and offer practical advantages, such as low production costs and excellent chemical stability, making them durable in various environments. With a high absorption coefficient of approximately 10^5 ^cm⁻¹, they effectively capture sunlight and optimize charge movement in solar cells. Additionally, they are resilient to moisture, light exposure, and thermal fluctuations, making them suitable for real-world applications^5^. In 2023, N. Thakur et al. conducted advanced simulations of BaZrSe_3 _solar cells using SCAPS-1D software, achieving an impressive PCE of 19.17%^16^. While this result marks a significant milestone, it also highlights the potential for further enhancement in device performance. This presents an exciting opportunity for the research community to not only optimize the efficiency of BaZrSe_3_ solar cells but also to explore the properties of other chalcogenide perovskites. However, it is essential to note that experimental investigations into these materials can be resource-intensive and time-consuming. Such studies often rely on sophisticated and costly characterization techniques, hindering progress. Therefore, developing theoretical models and comprehensive guidelines is essential to streamline future experimental efforts. These approaches can make research more efficient and effective, allowing for exploring the vast potential of these innovative absorbers for solar energy applications.

A diverse range of software tools is available for modelling the characteristics of solar cells, allowing researchers to explore the intricate relationship between structural parameters and performance outcomes. The most widely used software are SETFOS, SILVACO-ATLAS, COMSOL, Wx-AMPS, and SCAPS-1D^17–19^. This research specifically utilized SCAPS-1D (version 3.3.10), a sophisticated one-dimensional, steady-state simulation tool developed by Professor Marc Burgelman at Ghent University in Belgium^20^. SCAPS-1D stands out for its user-friendly interface and cost-free accessibility, making it an appealing option for academic and industrial research. The software supports the analysis of solar cell structures with up to seven layers, providing a platform for comprehensive batch analyses that yield reliable results with experimental data. Researchers can adjust essential parameters such as bandgap, carrier concentration, defect density, electron affinity, density of states (DOS), mobility, etc., allowing for extensive customization to match specific research needs. Additionally, SCAPS-1D features a variety of illumination options, including the standard AM0, AM1.5G, and monochromatic light sources^21–23^. This flexibility enhances its capability to conduct an in-depth evaluation of solar cell performance under different operational conditions. At its core, SCAPS-1D operates by solving fundamental semiconductor equations, including Poisson’s, carrier continuity, and drift-diffusion equations. This process facilitates the accurate calculation of current within defined limits based on the structural configuration of the solar cell and the tailored input material parameters, ultimately leading to a more nuanced understanding of solar energy conversion efficiency^24,25^.

This study introduces novel chalcogenide perovskite absorbers: CaZrSe_3_, BaZrSe_3_, CaHfSe_3_, and BaHfSe_3_. We designed devices with the structure FTO/TiO_2_/absorbers/NiO/Au to evaluate their potential for use in PVs using SCAPS-1D. Our analysis extensively examines the effects of carrier concentration, defect density, and the thickness of the TiO_2_ ETL, NiO HTL, and absorbers on solar cell performance, highlighting essential material properties. We also discuss the significant impact of defects at the ETL/absorber and HTL/absorber interfaces. Our research encompasses various analyses, including current density-voltage (J-V) measurements, capacitance-frequency (C-F) characteristics, recombination profiles, electric field distribution, energy band diagrams, and quantum efficiency (QE), to enhance our understanding of solar cell functionality. Finally, we assess the effects of series resistance, shunt resistance, and operating temperature. We believe that our findings will significantly contribute to the development of novel, cost-effective, non-toxic, and highly efficient solar cells based on CaZrSe_3_, BaZrSe_3_, CaHfSe_3_, and BaHfSe_3_ absorbers within the PV community.

## Computational approach and device overview

The SCAPS-1D is a well-established computational software developed by Mark Burgelman at the University of Ghent in Belgium. It is specifically designed to predict the performance of solar cells, focusing on the characteristics of individual layers and interfaces. This software offers several advantages, including the capability to analyze performance across up to seven layers^26^. Additionally, SCAPS-1D allows for the examination of key output parameters such as open circuit voltage (V_OC_), short circuit current density (J_SC_), fill factor (FF), and PCE for each layer. Its functionalities are enabled by solving three core equations: Poisson Eq. (1), continuity Eq. (2**)** and Eq. (3), and charge transport Eq. (4**) **and Eq. (5), which govern the behavior of charge carriers^21,23,27^;

1 $$\:\frac{{\partial\:}^{2\:}\phi\:\left(x\right)}{\partial\:{x}^{2}}=\:\frac{q}{\epsilon\:}\:\left(n\left(x\right)-p\left(x\right)-{N}_{D}^{+}\left(x\right)+{N}_{A}^{-}\left(x\right)-{p}_{t}\left(x\right)+{N}_{t}\left(x\right)\right)$$

2 $$\:\frac{\partial\:n}{\partial\:t}=\frac{1}{q}\frac{\partial\:{J}_{n}}{\partial\:x}+\left({G}_{n}-{R}_{n}\right)$$

3 $$\:\frac{\partial\:p}{\partial\:t}=-\frac{1}{q}\frac{\partial\:{J}_{p}}{\partial\:x}+\left({G}_{p}-{R}_{p}\right)$$

4 $$\:{J}_{n}=q{D}_{n}\frac{\partial\:n}{\partial\:x}-q{\mu\:}_{n}n\frac{\partial\:\phi\:}{\partial\:x}$$

5 $$\:{J}_{p}=q{D}_{p}\frac{\partial\:p}{\partial\:x}-q{\mu\:}_{p}p\frac{\partial\:\phi\:}{\partial\:x}$$

Herein, q stands for the elemental charge, ε represents the dielectric constant, p denotes the concentrations of holes, n signifies the concentration of electrons, $\:{N}_{D}^{+}$ represents the concentrations of the donor, $\:{N}_{A}^{-}$ stands for the concentration of acceptor-type dopants, G_n_ signifies the generation rates of electrons, G_p_ represents the generation rate of holes, R_n_ depicts the recombination rates of electrons, R_p_ denotes the recombination rates of holes, φ embodies the electric potential, D_n_ represents the coefficients of electron diffusion, D_p_ signifies coefficient of hole diffusion, J_p_ denotes the densities of the hole, J_n_ represents the density of electron current, µ_n_ stands for electron mobility and µp signifies holes mobility.

**Fig. 1:**
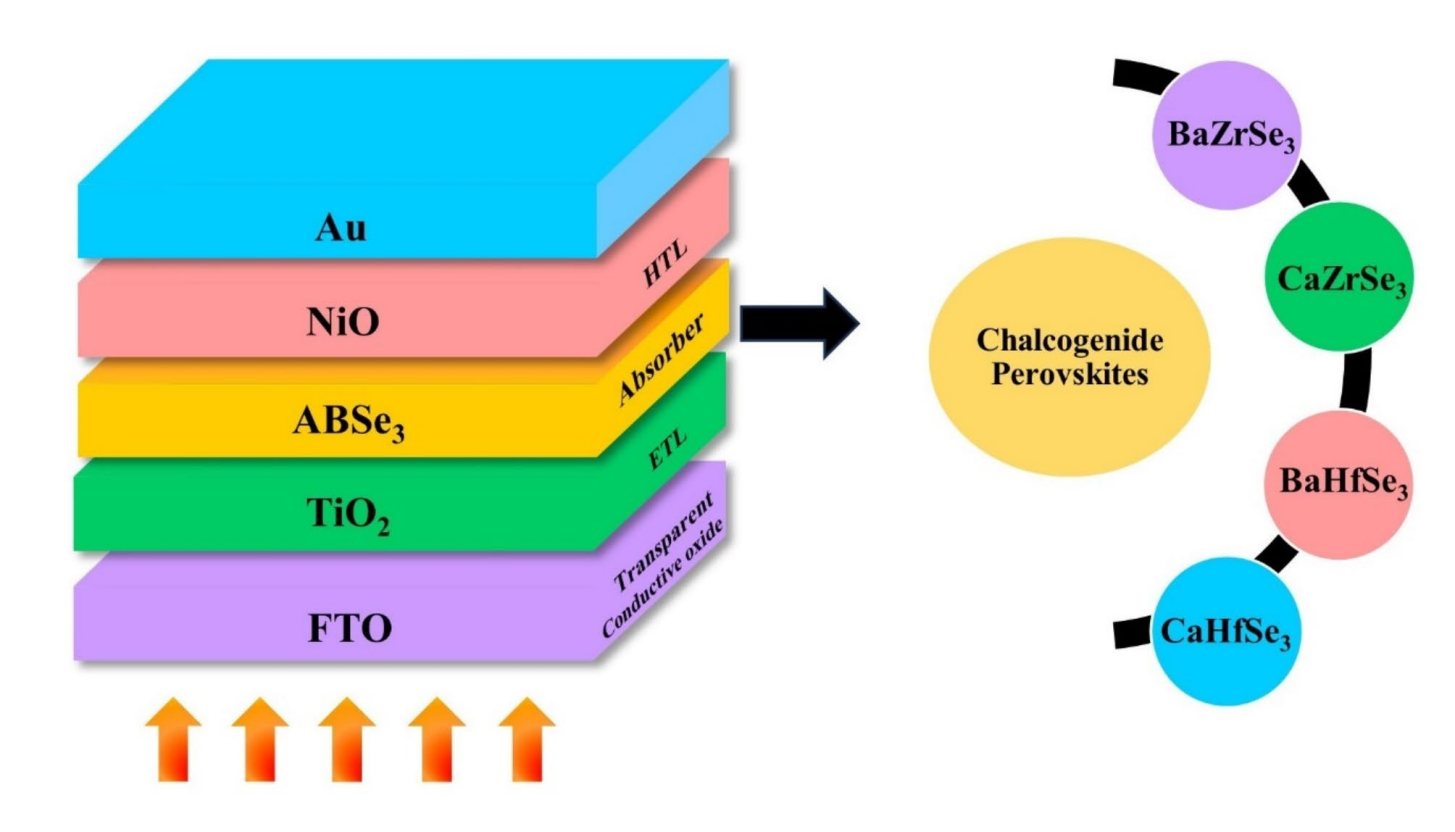
Schematic representation of novel chalcogenide perovskite solar cells.

**Fig. 2:**
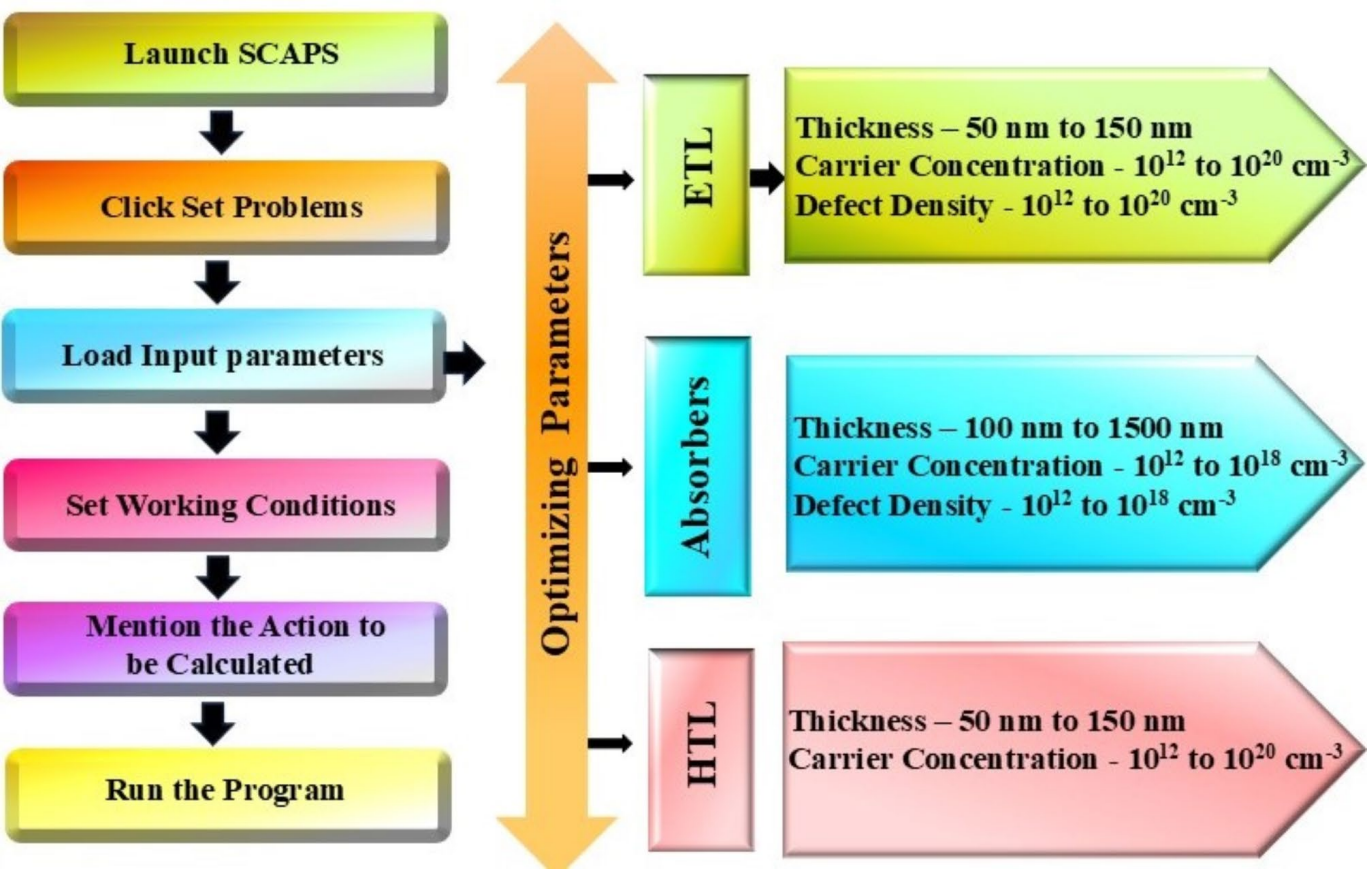
Flowchart representation of simulation process and optimization parameters of novel chalcogenide perovskite solar cells.

In this study, we conducted a detailed investigation into the performance of novel chalcogenide solar cells using SCAPS-1D. The solar cells featured innovative chalcogenide perovskite absorbers: CaZrSe_3_, BaZrSe_3_, CaHfSe_3_, and BaHfSe_3_. We adopted a superstrate device configuration, which is structured as follows: FTO/TiO_2_/A (Ca, Ba) B (Zr, Hf) Se_3_/NiO/Au as illustrated in Fig. 1 and flow chart of simulation in Fig. 2. The parameters for each layer were set based on established literature, detailed comprehensively in Table 1. bandgap (E_g_), affinity (χ), dielectric permittivity ((ε_r_), effective density of states in the conduction and valence bands (N_c_, N_v_), electron and hole mobilities (µ_n_, µ_p_), donor and acceptor concentrations (N_D_, N_A_) and defect densities (N_t_). We conducted the simulations at 300 K under AM 1.5G solar spectrum irradiance. The work functions were − 4.07 eV for Au and − 5.1 eV for FTO. Additionally, the surface recombination velocities for both electrons and holes were set at 1 × 10^7^, as indicated in Table 2. To simulate realistic solar cell conditions, we introduced neutral defects at the Absorber/HTL and ETL/Absorber interfaces, as detailed in Table 2.

Initially, the study concentrated on designing solar cells using the parameters outlined in Table 1. Subsequently, the research explicitly investigated the effects of varying the thickness (ranging from 50 nm to 150 nm), carrier concentration (from 10^12^ cm^−3^ to 10^20^ cm^−3^), and defect density (also from 10^12^ cm^−3^ to 10^20^ cm^−3^) of the ETL. Additionally, the potential of chalcogenide perovskite absorbers such as CaZrSe_3_, BaZrSe_3_, CaHfSe_3_, and BaHfSe_3_ was assessed by altering their thickness (between 100 nm and 1500 nm), carrier concentration, and defect density within the same ranges as mentioned above. Furthermore, the role of HTL was explored by optimizing its thickness (from 50 nm to 150 nm), carrier concentration (from 10^12^ cm^−3^ to 10^20^ cm^−3^), and defect density (from 10^12^ cm^−3^ to 10^20^ cm^−3^). The impact of defects at both the ETL/Absorber and Absorber/HTL interfaces was also examined. Overall, the findings were supported by various measurements, including J-V, C-F, electric field distribution, recombination profiles, generation rate, energy band diagram and QE. The series resistance (R_S_), shunt resistance (R_sh_), and operating temperature were varied from 1 to 10 Ω cm², 1000 to 5000 and 300 to 480 K respectively for CaZrSe_3_, BaZrSe_3_, CaHfSe_3_, and BaHfSe_3_ solar cells.

**Table 1:** SCAPS input parameters for initial chalcogenide perovskite solar cells^16,23,28–35^.

Parameter	FTO	TiO_2_	CaZrSe_3_	BaZrSe_3_	CaHfSe_3_	BaHfSe_3_	NiO
**Thickness (nm)**	200	50	500	500	500	500	80
**E**_**g**_ **(eV)**	3.5	3.2	1.4	1.35	1.65	1.5	3.25
**χ (eV)**	4.0	4.0	3.8	3.8	3.7	3.8	1.8
**ε**_**r**_	9.0	9.0	11.0	11.0	11.0	11.0	11.75
**N**_**C**_ **(cm** ^**−3**^ **)**	2.2E + 18	2.2E + 18	2.2E + 18	2.2E + 18	2.2E + 18	2.2E + 18	2.0E + 18
**N**_**V**_ **(cm** ^**−3**^ **)**	1.8E + 19	1.8E + 19	1.8E + 19	1.8E + 19	1.8E + 19	1.8E + 19	1.8E + 19
**µ**_**n**_ **(cm** ^**2**^ **/Vs)**	2E + 1	2E + 2	2.8E-2	2.8E-2	7.6E-2	9.4E-2	8E + 0
**µ**_**h**_ **(cm** ^**2**^ **/Vs)**	1E + 1	1E + 1	5.9E-2	8.2E-2	3.4E-2	3.5E-2	2E + 0
**N**_**D**_ **(cm** ^**−3**^ **)**	1E + 20	1E + 17	0	0	0	0	0
**N**_**A**_ **(cm** ^**−3**^ **)**	0	0	1E + 18	1E + 18	1E + 18	1E + 18	1E + 16
**N**_**t**_ **(cm** ^**−3**^ **)**	1E + 15	1E + 15	1E + 15	1E + 15	1E + 15	1E + 15	1E + 15

**Table 2:** Parameters for front and back contact, as well as interface defects, in novel chalcogenide perovskite solar cells^26,36,37^.

Front and back contact parameters		
Contacts	Back metal contact properties (Au)	Front metal contact Properties (FTO)
Metal work function (eV)	5.10	4.07
Surface recombination velocity of electron (cm/s)	1.000 × 10^7^	1.000 × 10^7^
Surface recombination velocity of hole (cm/s)	1.000 × 10^7^	1.000 × 10^7^
**Interface defect parameter**		
**Parameters (unit)**	**A (Ca**,** Ba) B (Zr**,** Hf) Se**_**3**_**/TiO**_**2**_**interface**	**A (Ca**,** Ba) B (Zr**,** Hf) Se**_**3**_**/NiO interface**
Defect density (cm^−3^)	1.0 × 10^12^ cm^−2^	1.0 × 10^12^ cm^−2^
Defect type	neutral	neutral
Capture cross section for electrons (cm^2^)	1.0 × 10^−19^ cm^2^	1.0 × 10^−19^ cm^2^
Capture cross section for holes (cm^2^)	1.0 × 10^−19^ cm^2^	1.0 × 10^−19^ cm^2^
Energetic distribution	single	single
Reference for defect energy level E_t_	Above the highest valence band	Above the highest valence band
Energy level with respect to valence band maximum (eV)	0.6 eV	0.6 eV

## Results and discussion

### Initial device performance

The initial solar cell was designed with the structure of FTO/TiO_2_/ A (Ca, Ba) B (Zr, Hf) Se_3_/ NiO/ Au, as shown in Fig. 1. Simulations were carried out using the parameters listed in Table 1 and 2. The initial PV parameters for the novel solar cells, specifically CaZrSe_3_, BaZrSe_3_, CaHfSe_3_, and BaHfSe_3_, are presented in Table 3. Notably, these novel chalcogenide perovskite solar cells achieved PCEs of 9.94% for CaZrSe_3_, 10.14% for BaZrSe_3_, 8.20% for CaHfSe_3_, and 13.04% for BaHfSe_3_ absorbers. The performance of the solar cells is further improved through careful optimization of the ETL, absorber, and HTL material parameters. Detailed insights into these optimization processes are discussed in the following sections.

**Table 3:** Initial PV parameters of novel chalcogenide perovskite solar cells.

Solar cell configuration	V_OC_ (V)	J_SC_ (mA/cm^2^)	FF (%)	PCE (%)
FTO/ TiO2/CaZrSe_3_/NiO/Au	1.03	12.17	79.06	9.94
FTO/ TiO_2_/BaZrSe_3_/NiO/Au	0.98	13.04	78.85	10.14
FTO/ TiO_2_/CaHfSe_3_/NiO/Au	1.17	8.73	80.08	8.20
FTO/ TiO_2_/BaHfSe_3_/NiO/Au	1.13	13.88	82.57	13.04

### Impact of ETL’s thickness, carrier concentration, and defect density

**Fig. 3:**
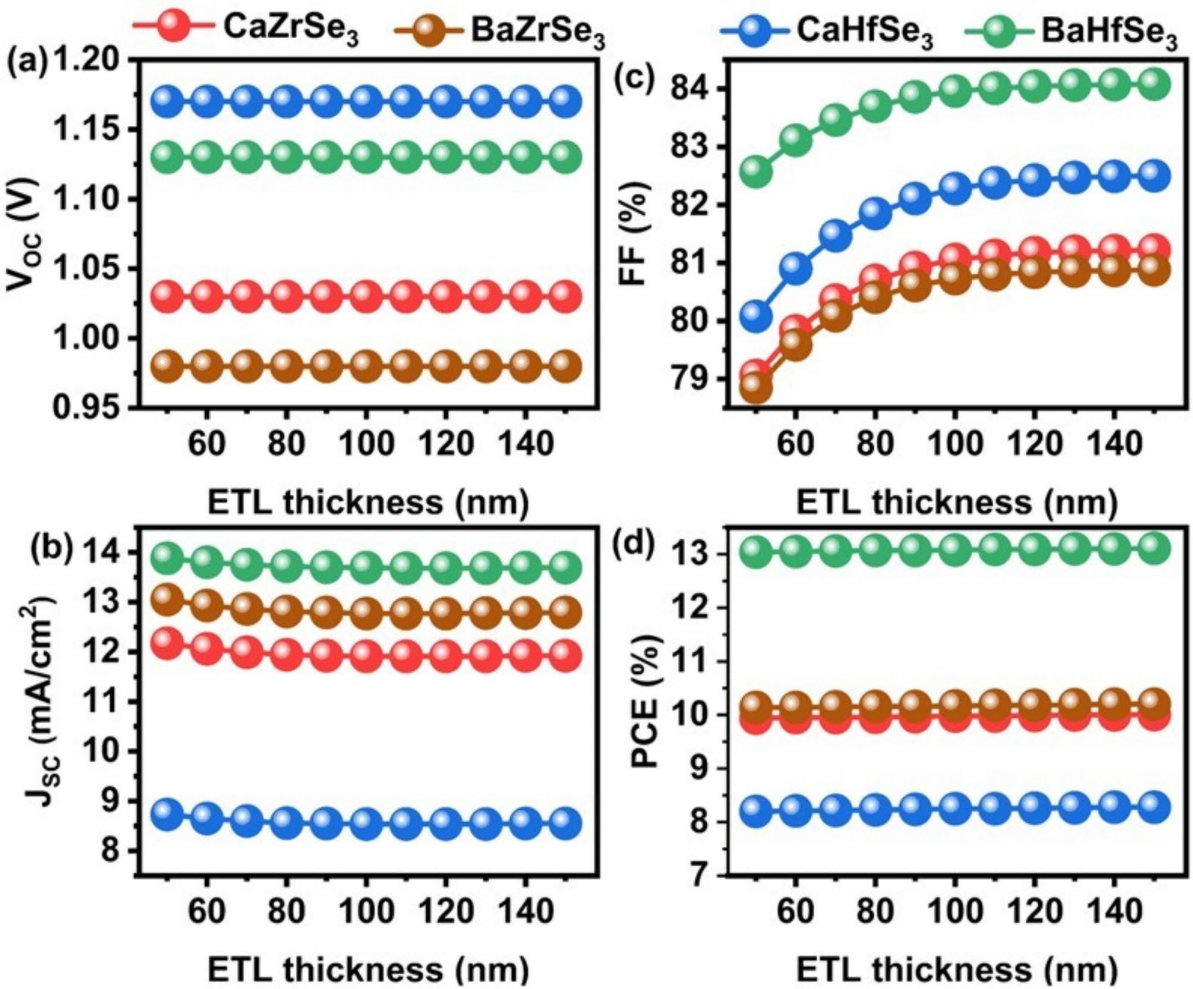
Changes in (**a**) V_OC_ (**b**) J_SC_ (**c**) FF and (**d**) PCE as a function of ETL’s thickness.

The thickness of the ETL plays a critical role in the transportation of electrons, enhancing light transmission, and preventing recombination within solar cells^21,23,38^. In this context, the ETL thickness varied from 50 nm to 150 nm, and its impact on PV parameters is illustrated in Fig. 3 (a-d). Notably, the V_OC_ remains unchanged across this range, while the FF increases from 79.06%, 78.85%, 80.08%, and 82.57 to 81.22%, 80.88%, 82.50%, and 84.08% for CaZrSe_3_, BaZrSe_3_, CaHfSe_3_, and BaZrSe_3_ based solar cells respectively. This improvement can be attributed to the reduced R_S_ and recombination rates at the Absorber/ETL interface. Consequently, there is a slight increase in the PCE, rising from 9.84 to 10.01%, 10.14 to 10.20%, 8.20 to 8.28%, and 13.04 to 13.11% for CaZrSe_3_, BaZrSe_3_, CaHfSe_3_, and BaZrSe_3_-based solar cells, respectively. However, a slight decrease in Jsc is observed as the thickness increases from 50 nm to 150 nm. This decline is primarily attributed to the transparent properties of the ETL. Typically, it is preferable to keep the n-type layer thinner than the p-type layer. This approach allows photons to reach the upper layers without being absorbed, which prevents them from being converted into electron-hole pairs that are then separated by built-in potential. Furthermore, a thicker ETL tends to absorb some light, thereby slowing down charge generation and collection. This results in absorption losses and reduced transmittance. The relationship between ETL thickness and transmittance is described by Eq. (6**)**^21,23^.

6 $$\:{\alpha\:}_{e}=\:\frac{1}{{d}_{e}}\text{ln}\frac{1}{{T}_{e}}$$

Here, d_e_, T_e_, and α_e_ represent the thickness, transmittance, and absorption coefficient, respectively. Based on observations and experimental challenges, an ideal thickness of 50 nm is chosen for CaZrSe_3_, BaZrSe_3_, CaHfSe_3_, and BaZrSe_3_-based solar cells.

**Fig. 4:**
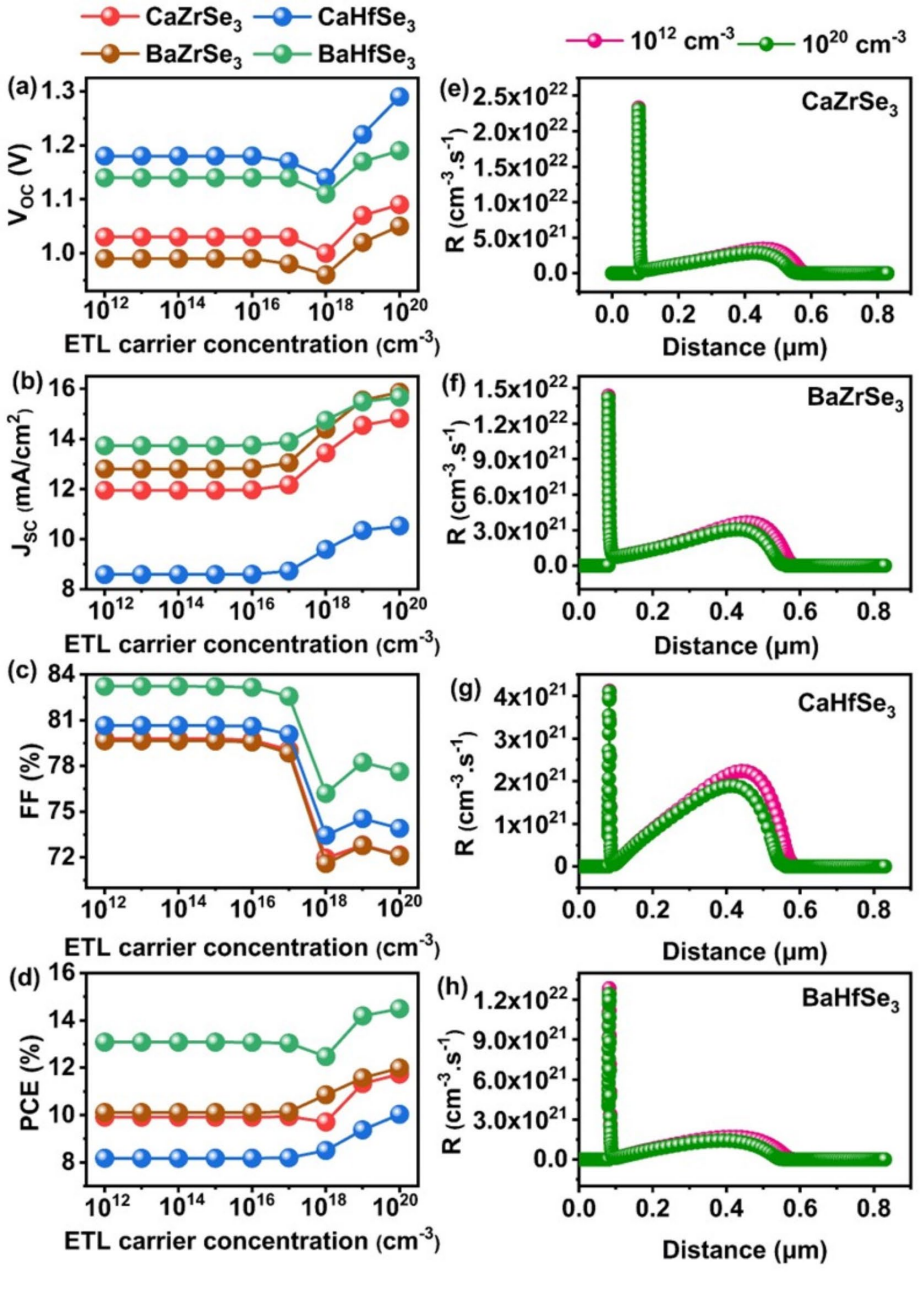
Changes in (**a**) V_OC_ (**b**) J_SC_ (**c**) FF (**d**) PCE and (**e**-**h**) Recombination rates as a function of ETL’s carrier concentration.

The carrier concentration of the ETL is varied from 10^12^ cm^−3^ to 10^20^ cm^−3^ to investigate its influence on the PV parameters, as shown in Fig. 4(a-d). The Voc remained relatively constant at 1.03 V, 0.99 V, 1.18 V, and 1.13 V for the CaZrSe_3_, BaZrSe_3_, CaHfSe_3,_ and BaHfSe_3_-based solar cells, respectively, until the carrier concentration reached 10^16^ cm^−3^. Beyond this point, V_OC_ began to decline. This decrease indicates a reduction in quasi-Fermi level splitting as the carrier concentration increases^39^. Similarly, the FF remained constant across all solar cells up to 10^16^ cm^−3^, followed by a slight decrease at higher concentrations. Interestingly, when the carrier concentration exceeded 10^16^ cm^−3^, there was a gradual increase in J_SC_, which ultimately led to an improvement in PCE. The PCE increased from 9.91 to 11.73% for CaZrSe_3_, from 10.11 to 11.99% for BaZrSe_3_, from 8.18 to 10.03% for CaHfSe_3_, and from 13.08 to 14.49% for BaHfSe_3_. This enhancement is attributed to the increase in built-in potential and conductivity in the solar cells with higher carrier concentrations^21,23^. Generally, at lower carrier concentrations, the holes mainly occupy the interface states, which act as traps for electrons, thereby impeding the flow of photogenerated charge carriers. Conversely, an increase in carrier concentration lowers the barrier height at the ETL/Absorber interface^21,27,40,41^. Xu et al. demonstrated that a higher carrier concentration generates deep energy levels at the interfaces, which reduces non-radiative recombination and enhances solar cell performance. As shown in Fig. 4(e-h), when the carrier concentration is high (at 10^20^ cm^−3^), all the solar cells exhibit lower recombination rates compared to those with low carrier concentration (10^12^ cm^−3^). Therefore, an optimal carrier concentration is crucial for ensuring proper band alignment, generating a substantial electric field that facilitates efficient charge carrier transport, reducing interface recombination rates, and consequently improving solar cell performance^42^. As a result, an ideal carrier concentration of 10^20^ cm^−3^ is selected for all the solar cells.

**Fig. 5:**
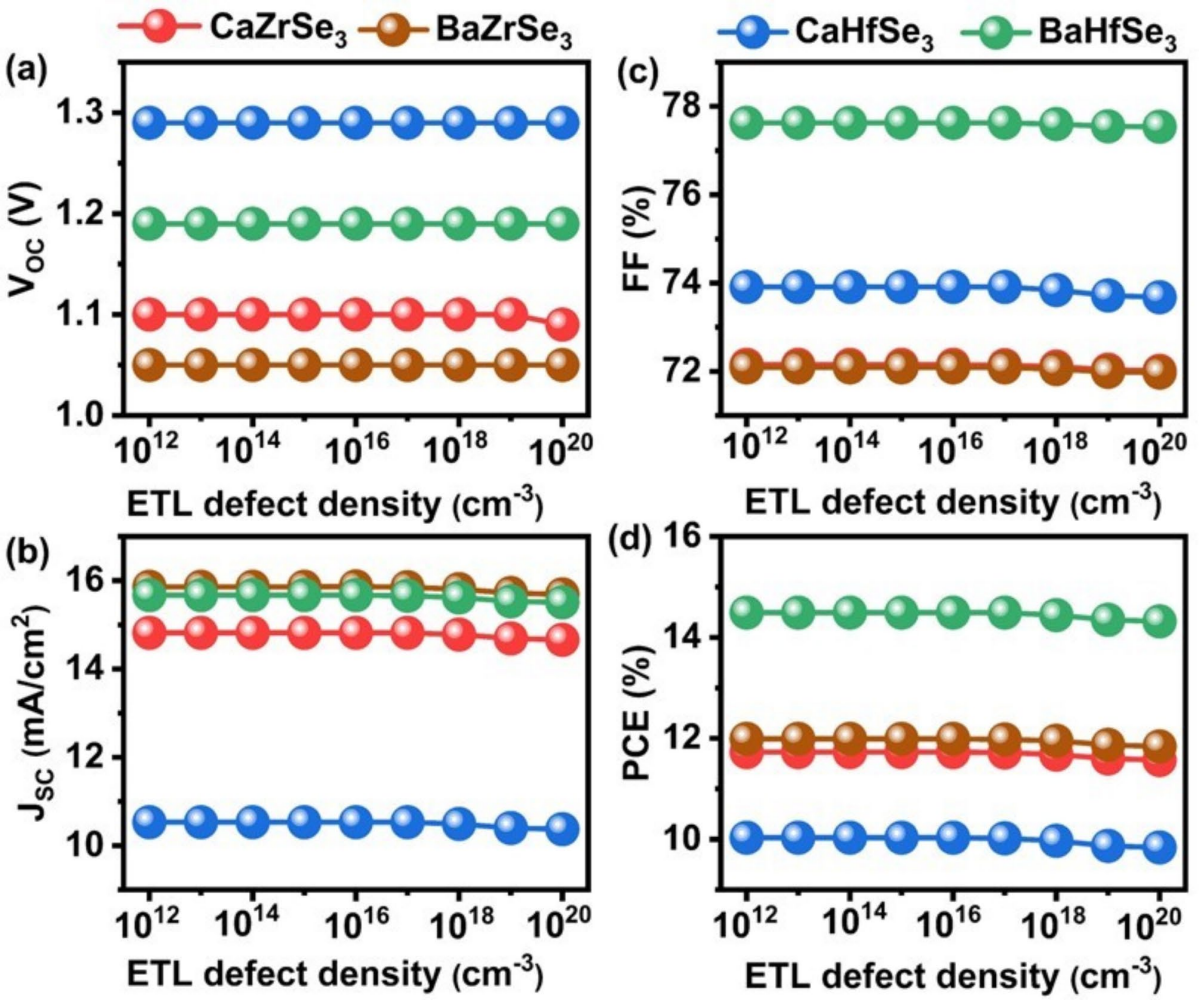
Changes in** (a)** V_OC_ **(b)** J_SC_ **(c)** FF and **(d) **PCE as a function of ETL’s defect density.

Defects in materials create additional pathways for non-radiative recombination, converting light into heat instead of electricity. Specifically, these recombination centers trap photogenerated carriers, preventing them from reaching the terminals and thereby reducing their lifespan^23^. Consequently, minimizing defects is essential for improving device performance. To investigate their impact, we varied the defect density from 10^12^ cm^−3^ to 10^20^ cm^−3^, as shown in Fig. 5(a-d). Across all solar cells, the PV parameters remained stable within the range of 10^12^ cm^−3^ to 10^16^ cm^−3^, but exhibited a slight decline as defect density increased further. Specifically, the PCE decreased from 11.73 to 11.57% for CaZrSe_3_, from 11.99 to 11.84% for BaZrSe_3_, from 10.03 to 9.84% for CaHfSe_3_, and from 14.49 to 14.32% for BaHfSe_3 _solar cells. This drop in performance is attributed to the rising number of defect states, which act as barriers for charge carriers, thereby enhancing recombination within the solar cells^43^. Nevertheless, it is important to note that the reduction in performance is minimal specifically, 0.16%, 0.15%, 0.19%, and 0.17% for CaZrSe_3_, BaZrSe_3_, CaHfSe_3,_ and BaHfSe_3_-based solar cells, respectively. This indicates that the influence of ETL defect density on solar cell performance is negligible. Therefore, an optimal defect density of 10^16^ cm^−3^ is chosen for the subsequent simulations.

### Impact of absorber’s thickness, carrier concentration and defect density

**Fig. 6:**
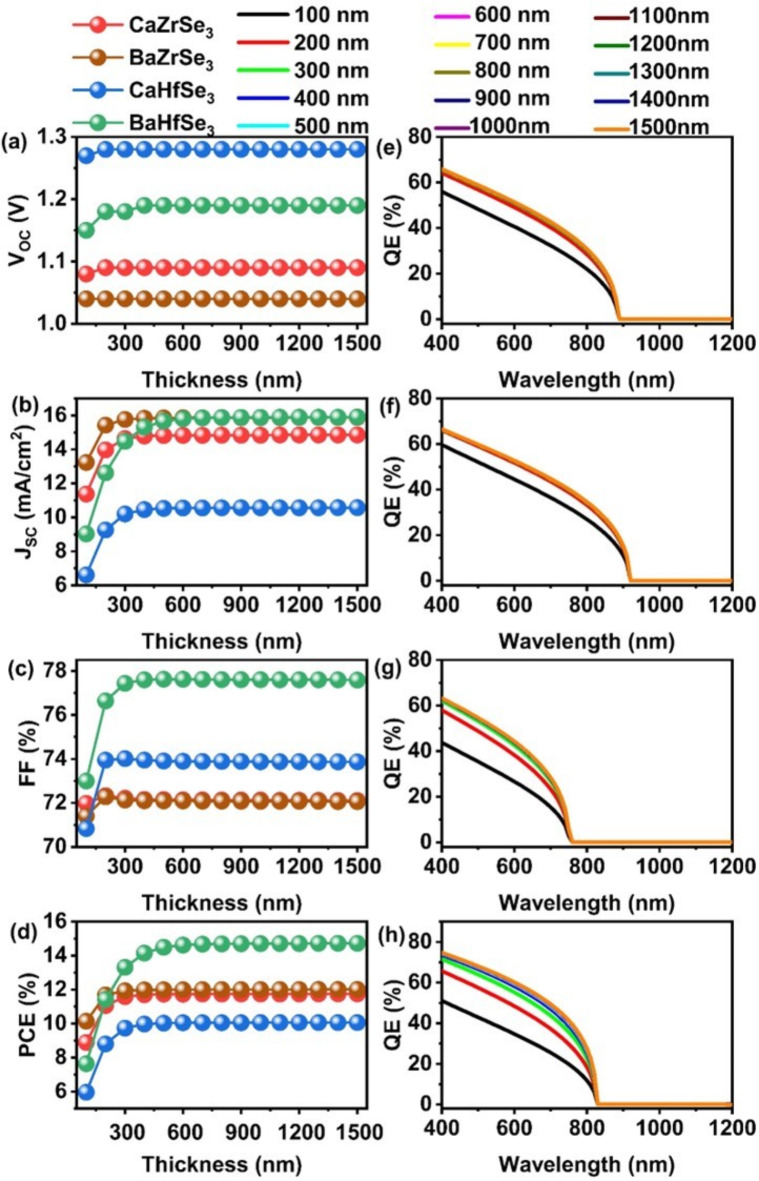
Changes in (**a**) V_OC_ (**b**) J_SC_ (**c**) FF (**d**) PCE and (**e**-**h**) QE as a function of absorber’s thickness.

The thickness of the absorber has a significant impact on the performance of solar cells^44^. To determine the optimal thickness, we varied it from 100 nm to 1500 nm for all absorbers, as illustrated in Fig. 6(a-d). As the thickness increases from 100 nm to 500 nm, the J_SC_ shows a substantial increase from ~ 6 mA/cm^2^ to 15 mA/cm^2^ across all solar cells. This leads to a rise in the PCE from 8.89 to 11.75% for CaZrSe_3_, from 10.14 to 12.01% for BaZrSe_3_, from 5.96 to 10.06% for CaHfSe_3_, and from 7.64 to 14.72% for BaHfSe_3_. The improvement in performance occurs because thinner absorbers are unable to effectively absorb photons with longer wavelengths, resulting in lower photon absorption and insufficient generation of charge carriers^45^. As the thickness increases, the ability to absorb photons improves, leading to enhanced charge carrier generation and a marked boost in solar cell performance^46^. However, an interesting trend is observed in the PCE once the thickness exceeds 500 nm, where the improvements become marginal, only increasing by about 0.03% across all solar cells. Initially, the increase in thickness from 100 nm to 500 nm results in substantial gains in PCE: 2.84% for CaZrSe_3_, 1.85% for BaZrSe_3_, 4.07% for CaHfSe_3_, and 6.85% for BaHfSe_3_. When the thickness goes beyond 500 nm, the distance that charge carriers must travel to reach their respective contacts becomes significant^45,47^. Consequently, many of these carriers recombine due to their limited diffusion lengths compared to the thickness of the absorber, leading to a saturation in solar cell performance^21^. This phenomenon is further supported by QE measurements (Fig. 6 (e-h)), which show that the increase in absorption is 5.83%, 4.77%, 7.3%, and 11.93% for CaZrSe_3_, BaZrSe_3_, CaHfSe_3_, and BaHfSe_3_ solar cells, respectively, within the thickness range of 100 nm to 500 nm. Beyond this, the absorption increases by about 0.4% with additional thickness. On the other hand, the V_OC _improves up to 300 nm, after which it stabilizes for all solar cells. This initial enhancement is attributed to increased quasi-Fermi level splitting from greater charge carrier generation, while the later stabilization is a result of higher dark saturation currents and recombination rates associated with greater thickness^21^. The FF shows a similar trend, increasing until 300 nm before stabilizing across all solar cells due to increased R_S_ in thicker absorbers. Recent studies on chalcogenide perovskite solar cells indicate that when the absorber layer thickness exceeds 500 nm, there is a notable decrease in the built-in electric field. This reduction adversely affects effective carrier extraction, ultimately lowering the device’s PCE.

Research by Sun et al. examined the optoelectronic properties of CaTiS_3_, BaZrS_3_, CaZrS_3_ and CaHfS_3 _chalcogenide perovskite materials through DFT^5^. Their findings suggested that light absorption and carrier dynamics are achievable with absorber layer thicknesses significantly below 1000 nm, providing greater flexibility in design decisions. A pivotal study conducted by Nishigaki et al. involved the synthesis of several chalcogenide compounds, such as BaZrS_3_, Ba(Zr, Ti)S_3_, and BaZr(S, Se)_3_. This research emphasized the significance of absorber layer thickness, revealing that an optimal thickness of approximately 500 nm is essential for maximizing light absorption, a critical factor for developing high-efficiency solar cells^48^. Additionally, Himanshu et al. utilized SCAPS-1D theoretical simulations to evaluate the performance of BaZrS_3 _as an absorber layer. Their results demonstrated that a layer thickness of 500 nm can lead to impressive PCE, highlighting the material’s potential for practical applications^49^. Another significant contribution came from Swarnkar et al., who successfully synthesized various compounds, including BaZrS_3_, SrZrS_3_, and the layered perovskite Ba_3_Zr_2_S_7_^50^. They suggested that an absorber layer thickness of around 550 nm could maximize the PCE of solar cells, indicating a general consensus on the optimal thickness range. In summary, several reports advocate for absorber thicknesses between 500 nm and 1000 nm for chalcogenide perovskite solar cells to achieve high PCE^23,27,43,44^. Based on a thorough review of the collective evidence from these studies, an optimal thickness of 500 nm has been selected for CaZrSe_3_, BaZrSe_3_, CaHfSe_3_, and BaHfSe_3_-based solar cells, aligning well with the established best practices in the field.

The carrier concentration in the absorber layer is crucial for governing both charge transport and the overall stability of solar cells^21^. Therefore, determining the optimal carrier concentration is essential to achieve high-performance solar cells. While a higher carrier concentration can enhance certain properties, it also increases the risk of Auger recombination, which can hinder hole movement by causing significant recombination and impurity scattering within the absorber layer^51,52^. Consequently, optimizing the carrier concentration is vital for improving solar cell efficiency.

**Fig. 7:**
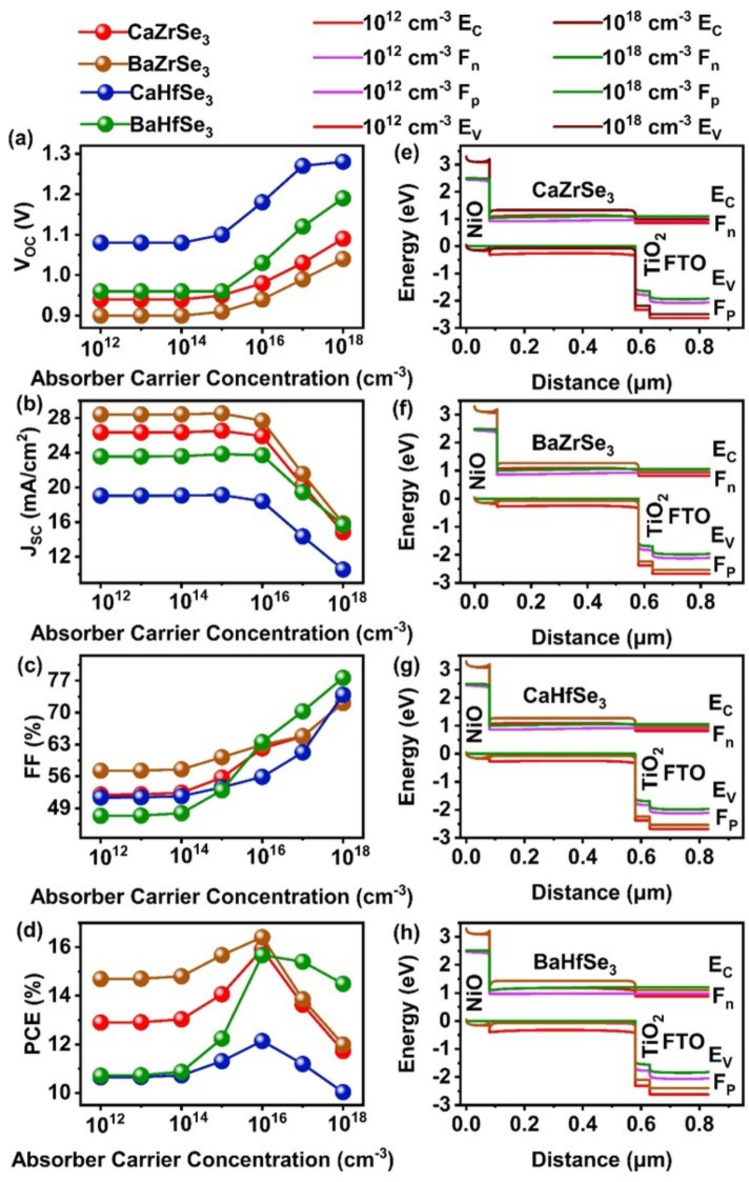
Changes in **(a)** V_OC_ **(b)** J_SC_ **(c)** FF** (d)** PCE and **(e-h)** Energy band alignment as a function of absorber’s carrier concentration.

In our study, we varied the carrier concentration of CaZrSe_3_, BaZrSe_3_, CaHfSe_3_ and BaHfSe_3_ based absorbers from 10^12^ cm^−3^ to 10^18^ cm^−3^, as shown in Fig. 7(a-d). We observed that the PCE remained stable up to 10^14^ cm^−3^, followed by a sharp increase until 10^16^ cm^−3^; however, it declined with further increases in carrier concentration across all solar cells. Additionally, the V_OC_ and FF remained constant up to 10^14^ cm^−3 ^but showed significant improvement beyond that point. This enhancement can be attributed to the increased intrinsic capacity of the device, which allows for more efficient carrier extraction and accumulation at the contacts while minimizing recombination^27^. Moreover, varying the carrier concentration from 10^12^ cm^−3^ to 10^18^ cm^−3^ affects the energy band alignment and elevates the quasi-Fermi level splitting, resulting in higher V_OC _and improved overall performance^26^. To further investigate these effects, we extracted energy band diagrams from SCAPS-1D for carrier concentration ranging from 10^12^ cm^−3^ to 10^18^ cm^−3^, as depicted in Fig. 7(e-h). As the carrier concentration rises from 10^12^ cm^−3^ to 10^18^ cm^−3^, the energy bands of all layers shift upwards, bringing the valence band of the absorber closer to the hole quasi-Fermi level. This facilitates hole transport from the absorber to the HTL while simultaneously limiting electron transport and enhancing hole collection at the back contact^23,43^.

**Fig. 8:**
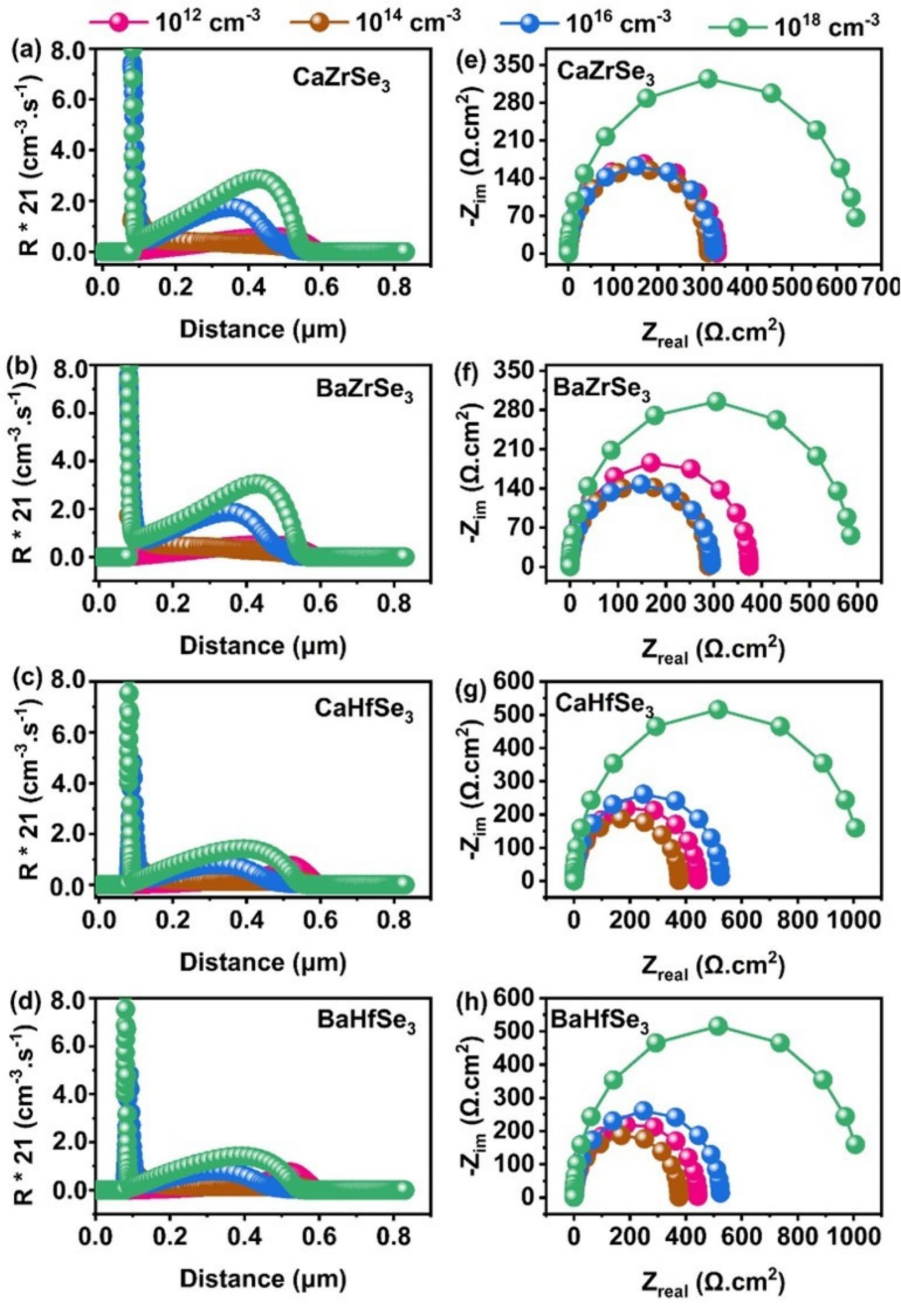
Changes in **(a-d)** Recombination rate and** (e-h)** Nyquist plot as a function of absorber’s carrier concentration.

Additionally, the electric field at the absorber/HTL interface intensifies with increased carrier concentration. However, we observed that the J_SC_ decreases when the carrier concentration exceeds 10^16^ cm^−3^. This decline can be attributed to the higher recombination rates caused by excessive carrier concentration, as demonstrated in Fig. 8(a-d). Significant recombination is evident beyond 10^16^ cm^−3^. Another factor contributing to the reduced J_SC_ at higher carrier concentration above 10^16^ cm^−3 ^is the shift of the depletion region at the ETL/absorber junction into the ETL, resulting in a narrower depletion width within the absorber. As a result, carriers with short-diffusion lengths and lifetimes are more likely to recombine, thereby lowering the J_SC_^21^. We obtained Nyquist plots to analyze the transport properties of charge carriers in all the solar cells, as illustrated in Fig. 8(e-h). Typically, Nyquist plots exhibit two semicircles: one representing recombination resistance at low frequencies and the other representing charge transfer resistance at high frequencies. However, in our case, we observed a single semicircle across the entire frequency range, indicating that charge transfer resistance predominates in all the devices. The smaller semicircle at 10^16^ cm^−3^ corresponds to high conductivity and efficient charge transfer. In contrast, higher carrier concentrates hinder charge transfer and degrade solar cell performance. Additionally, Meng et al. reported that chalcogenide perovskite films synthesized under S-rich/Zr-poor conditions exhibit strong p-type behavior with an optimal carrier concentration of 10^15^ cm^−3^. Conversely, films synthesized under S-poor/Zr-rich conditions display n-type behavior with high carrier concentrations exceeding 10^17^ cm^−3^, making them unsuitable as absorbers^53^. To achieve high PCE, it is recommended that the carrier concentration for chalcogenide perovskite solar cell materials stay below 10^17^cm^−3^^23,27,37,43,44,53^. After considering all relevant factors, we selected an optimal carrier concentration of 10^16^ cm^−3^ for CaZrSe_3_, BaZrSe_3_, CaHfSe_3_, and BaHfSe_3_-based solar cells, which falls within the suggested range.

**Fig. 9:**
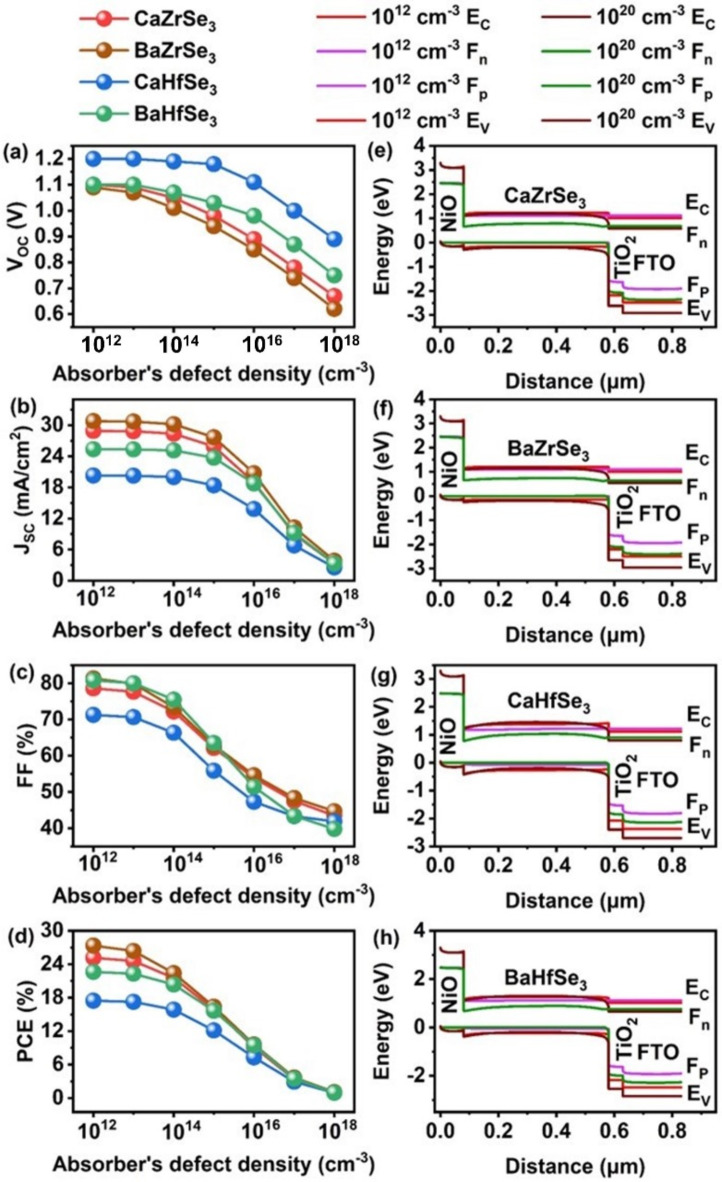
Changes in (**a**) V_OC_ (**b**) J_SC_ (**c**) FF (**d**) PCE and (**e**-**h**) Energy band alignment as a function of absorber’s defect density.

**Fig. 10:**
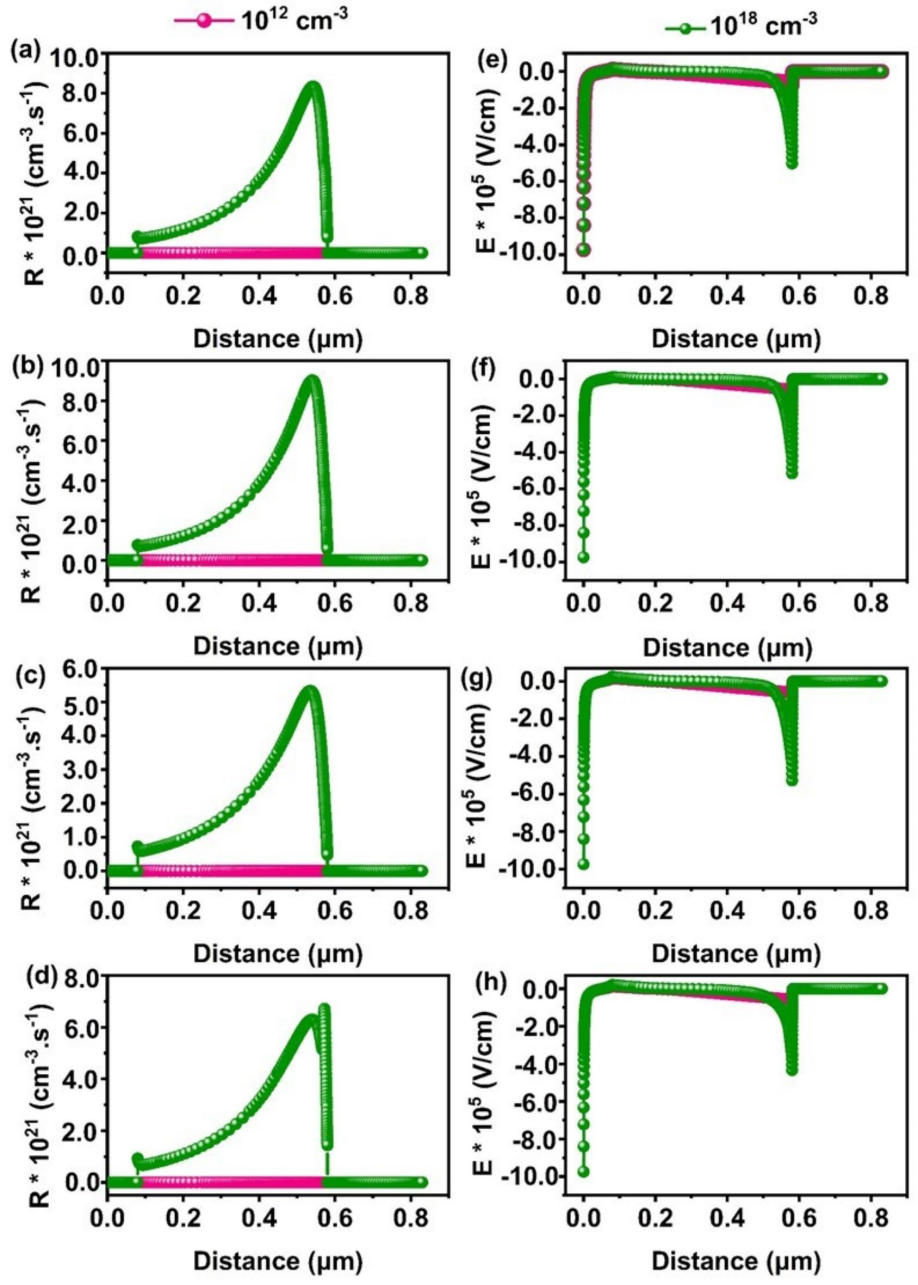
Changes in (**a-d**) Electric field and (**e-h**) Recombination rates as a function of absorbers defect densities.

Defects in solar cell absorbers typically arise from structural irregularities, non-stoichiometry, impurities, and fabrication technique^54^. Managing these absorber defects is crucial for achieving high efficiency in solar cells. To study the impact of absorber defect density on photovoltaic performance, we varied the defect density from 10^12^ cm^−3^ to 10^18^ cm^−3^ for CaZrSe_3_, BaZrSe_3_, CaHfSe_3_ and BaHfSe_3_ based solar cells. Figure 9(a-d) illustrates how the V_OC_, J_SC_, FF, and PCE respond to changes in the absorber’s defect density. Notably, all PV parameters significantly decrease as defect density increases. Specifically, V_OC_ and FF remain stable up to a defect density of 10^13^ cm^−3^, but they drop sharply beyond this threshold. Additionally, increasing defect density disrupts the energy band alignment and lowers the quasi-Fermi level splitting, resulting in reduced V_OC_ and negatively impacting solar cell performance. To further investigate these effects, we extracted energy band diagrams for defect densities of 10^12^ cm^−3^ and 10^18^ cm^−3^ using SCAPS-1D, as shown in Fig. 9(e-h). As defect density increases from 10^12^ cm^−3^ to 10^18^ cm^−3^, the energy bands of all layer’s shift downward at higher defect densities. This shift lowers the barrier at the ETL/absorber interface and reduces the quasi-Fermi level splitting, further degrading the V_OC _of all solar cell^55,56^. For instance, when the defect density rises from 10^12^ cm^−3^ to 10^18^ cm^−3^, the PCE declines dramatically from 25.17 to 0.96%, 27.35 to 1.08%, 17.47 to 0.96% and 22.63 to 0.99% for CaZrSe_3_, BaZrSe_3_, CaHfSe_3_ and BaHfSe_3_based solar cell respectively. This significant decrease in performance is primarily due to the increased recombination sites for photogenerated charge carriers, which reduces both their diffusion length and lifetime^21,23,26,44^. Figure 10**(**a-d) illustrates the recombination rate as a function of increasing defect density across all solar cells. As defect density increases, the recombination rate surges in the absorber region, particularly near the absorber/ETL junction, leading to a decline in overall performance. Interestingly, recombination at the absorber/NiO interface decreases with higher defect density. This reduction occurs because the increased recombination within the absorber region significantly lowers the density of photogenerated carriers throughout the bulk of the absorber and in the regions near the interface. As a result, fewer charge carriers reach the electrodes, thereby reducing the recombination rate at the absorber/NiO interface^57^. Moreover, Fig. 10(e-h) demonstrates that the electric field at the absorber/ETL interface weakens as defect density increases. This reduction in the electric field hampers the effective separation and collection of charge carriers, leading to further degradation in the overall performance of the solar cells. The decline in minority carrier density, along with the weakening of the built-in electric field at the p-n junction, significantly affects the overall performance of the solar cells. Despite these challenges, chalcogenide perovskites are considered defect-tolerant due to their high formation energy, which makes them less susceptible to deep-level defect^21^. Furthermore, Meng et al. reported that chalcogenide perovskite films synthesized under S-rich/Zr-poor conditions exhibit strong p-type behavior, while this composition also helps reduce defect density. This reduction occurs because of the higher formation energy associated with deep-level defects, resulting in fewer defects overall. It is crucial to adjust the elemental composition during the synthesis of chalcogenide perovskite films to achieve optimal carrier concentration and minimize the formation of deep-level defects^53^. Similarly, Carmen Baiano et al. controlled defect formation by optimizing stoichiometry and annealing in an oxygen-rich environment^58^. For achieving high PCE, the literature recommends a defect density of 10^10^ to 10^12^ cm^−3 ^for chalcogenide perovskite solar cell materials^23,27,43,44^. After evaluating all relevant factors, an optimal defect density of 10^12^ cm^−3^ has been selected for CaZrSe_3_, BaZrSe_3_, CaHfSe_3_, and BaHfSe_3_-based solar cells, which falls within the suggested range.

*Impact of HTL thickness and carrier concentration*.

**Fig. 11:**
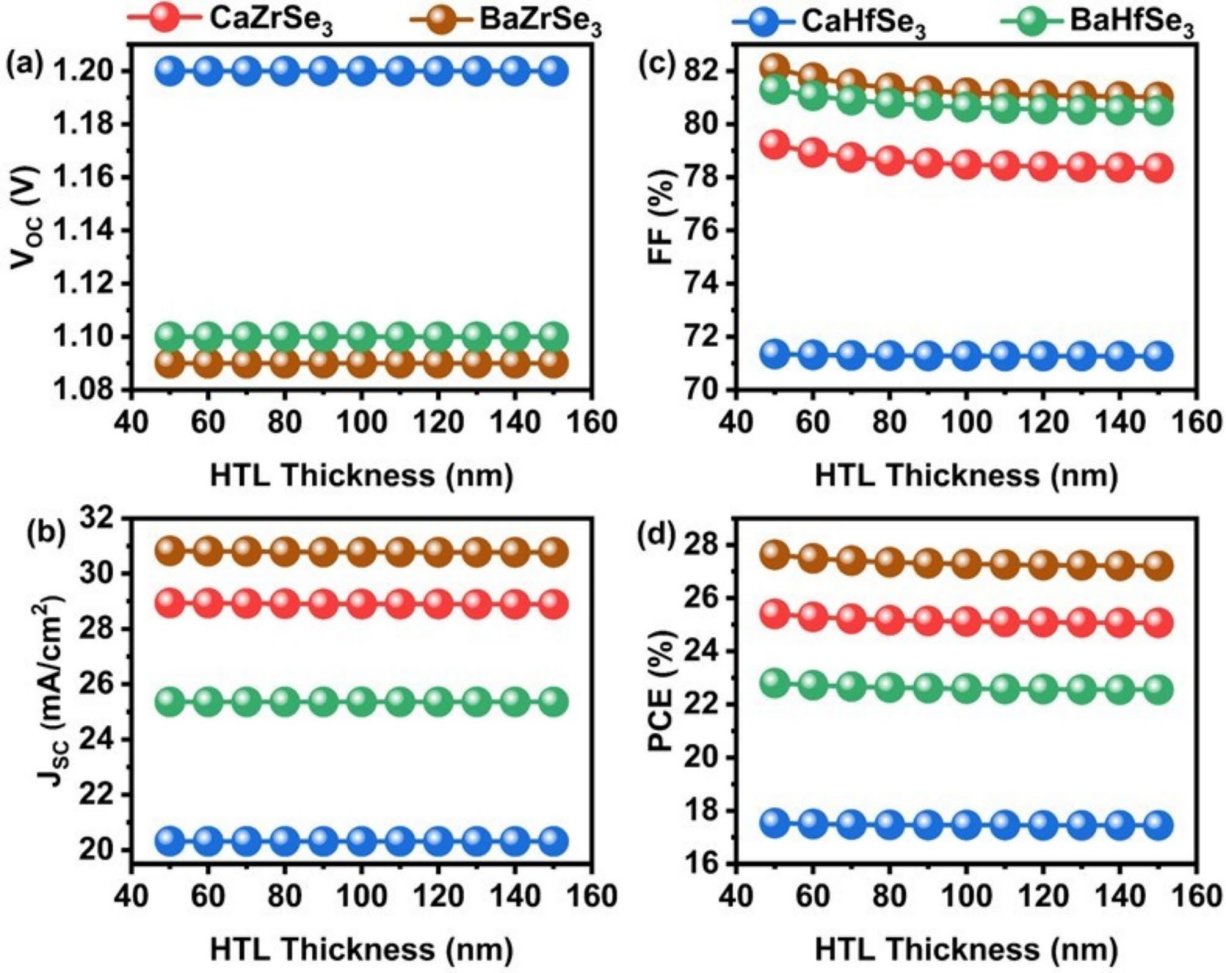
Changes in (**a**) V_OC_ (**b**) J_SC_ (**c**) FF and (**d**) PCE as a function of HTL thickness.

The HTL at the interface between the absorber and the back contact reduces the barrier for hole collection at the back contact, thereby decreasing interfacial recombination at the Absorber/HTL junction^21,58^. To investigate the impact of HTL thickness on solar cell performance, we varied the thickness from 50 nm to 150 nm across all solar cells (see Fig. 11(a-d)). Our observations indicate that changes in V_OC_, J_SC_, FF, and PCE relative to the HTL thickness are minimal, suggesting that the thickness has a limited effect on enhancing solar cell performance^31^. As a result, considering material costs and experimental factors, we selected an HTL thickness of 100 nm as the ideal thickness for subsequent simulations.

**Fig. 12:**
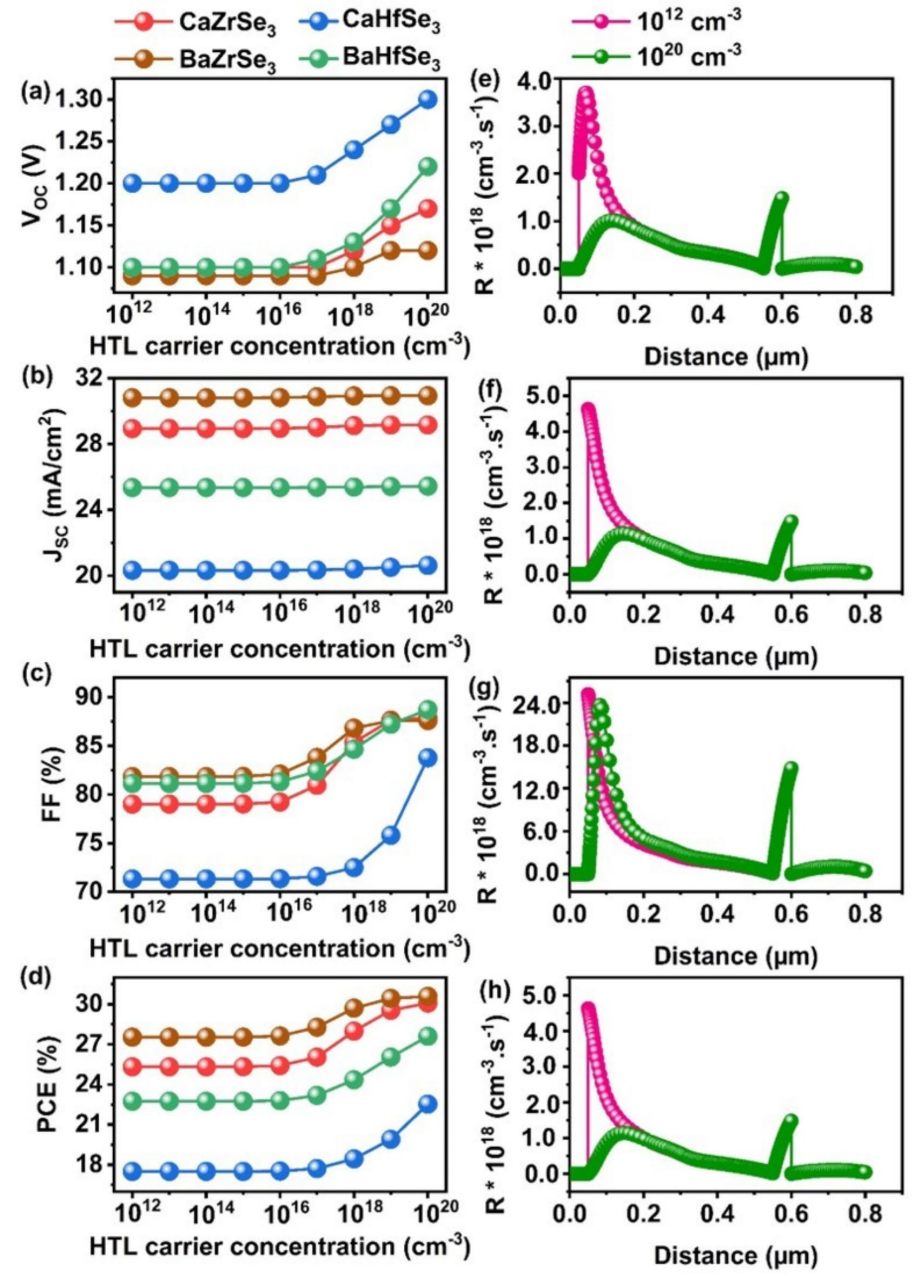
Changes in (**a**) V_OC_ (**b**) J_SC_ (**c**) FF (**d**) PCE and (**e**-**h**) Recombination rates as a function of HTL carrier concentration.

The impact of the HTL’s carrier concentration was studied by varying it from 10^12^ cm^−3^ to 10^20^ cm^−3^ for CaZrSe_3_, BaZrSe_3_, CaHfSe_3_, and BaHfSe_3_-based solar cells, respectively. Figure 12(a-d) illustrates how the PV parameters change with the HTL carrier concentration. Notably, the V_OC_ of all solar cells remains stable up to 10^16^ cm^−3^ and shows slight improvement beyond this threshold, while the J_SC_ remains unchanged throughout the entire range. The observed changes in V_OC _are linked to an increase in quasi-Fermi level splitting as the carrier concentration of the HTL increases^21,26^. In contrast, the FF values increase beyond 10^16^ cm^−3^, leading to an overall enhancement in PCE. This effect arises from the relationship between the carrier concentration of the HTL and the absorber. When the carrier concentration of the HTL is lower than that of the absorber, the energy bands remain unaffected, and therefore, the performance of the solar cells does not change until the threshold of 10^16^ cm^−3^ is reached. Additionally, barriers at the Absorber/HTL interface and in the back contact act as recombination centers, hindering the smooth flow of charge carriers towards their respective contacts, which further impacts the solar cell performance. Conversely, when the carrier concentration of the HTL exceeds that of the absorber, shifts in the energy bands occur, reducing the barriers at both interfaces. Moreover, the recombination rate at the Absorber/HTL interface decreases significantly with an increase in carrier concentration for all solar cells, as shown in Fig. 12(e-h). This improvement leads to increased transport efficiency, conductivity, and built-in potential within the solar cells^27^. Ultimately, the highest PCEs achieved were 30.08% for CaZrSe_3_, 30.58% for BaZrSe_3_, 22.53% for CaHfSe_3_, and 27.60% for BaHfSe_3_-based solar cells. In summary, these findings underscore the critical importance of HTL carrier concentration in enhancing solar cell performance.

### Impact of interface defect density on ETL/Absorber and Absorber/HTL

Interface defects are a common occurrence during the fabrication of solar cells due to structural imperfections^59^. These defects significantly enhance the recombination of charge carriers at the interface, adversely affecting solar cell performance. This highlights the importance of analyzing their influence and determining an optimal defect density for experimental fabrication. In this study, all simulations were based on a neutral interface defect density of 10^12^ cm^−3^ at the ETL/Absorber and Absorber/HTL interfaces for all solar cells. To investigate the impact on PV parameters, we varied the interface defect density from 10^12^ cm^−3^ to 10^20^ cm^−3^ for the ETL/Absorber interface and from 10^9^ cm^−3^ to 10^20^ cm^−3^ for the Absorber/HTL interface.

**Fig. 13:**
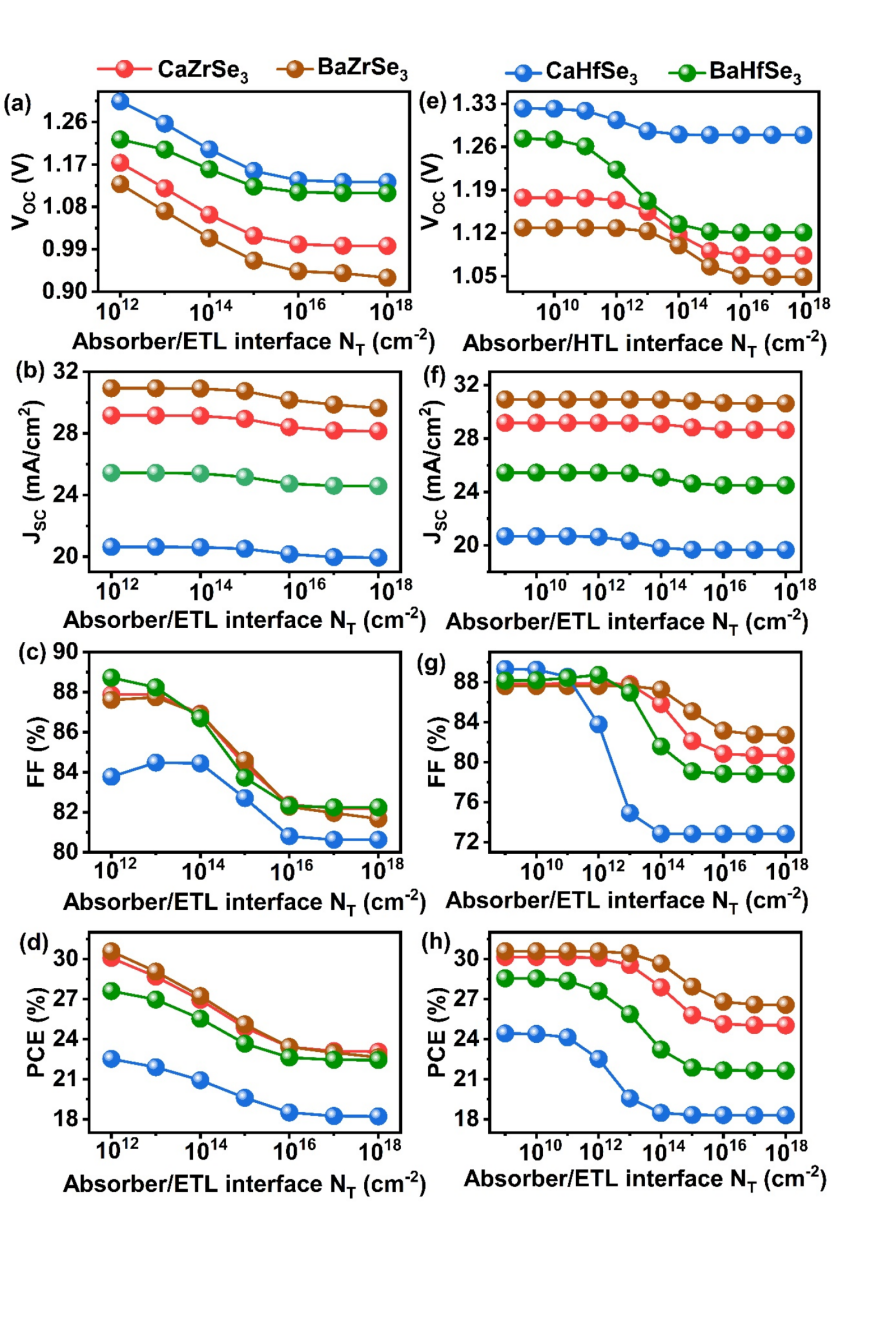
Changes in V_OC_, J_SC_, FF, and PCE as a function of (**a-d**) ETL/Absorber and (**e-h**) Absorber/HTL interface defects.

As shown in Fig. 13(a-d), the V_OC_ declines significantly with an increase in interface defects from 10^12^ cm^−3^ to 10^20^ cm^−3^. Meanwhile, the J_SC_ remains stable up to 10^14^ cm^−3^ before decreasing. Conversely, a marked drop in FF is observed, with values ranging from 87.88%, 87.61%, 83.78%, and 88.24% down to 82.19%, 81.68%, 80.63%, and 82.24% for CaZrSe_3_, BaZrSe_3_, CaHfSe_3_ and BaHfSe_3_-based solar cells, respectively. This leads to a significant reduction in PCE, indicating that FF plays a crucial role in determining solar cell performance at the ETL/Absorber interface. The decrease in PCE is approximately 7.01%, 7.94%, 4.31%, and 5.16% for CaZrSe_3_, BaZrSe_3_, CaHfSe_3_ and BaHfSe_3_-based solar cells, respectively, due to increased trap-assisted recombination at the ETL/Absorber interfaces, which impedes electron flow toward the front contact^59,60^. Given these results, an ideal defect density of 10^12^ cm^−3^ is recommended for the optimal performance of CaZrSe_3_, BaZrSe_3_, CaHfSe_3,_ and BaHfSe_3_-based solar cells. Next, we assessed the Absorber/HTL interface defect density by varying it from 10^9^ cm^−3^ to 10^20^ cm^−3^, as illustrated in Fig. 13 (e-h). Here, all solar cell parameters remain relatively stable up to 10^12^ cm^−3^ but decline beyond this threshold. Notably, PCE experiences a drastic drop from 30.16%, 30.59%, 22.53%, and 27.60–25.04%, 26.57%, 18.31%, and 21.64% for CaZrSe_3_, BaZrSe_3_, CaHfSe_3_ and BaHfSe_3_-based solar cells, respectively. This decline in performance can be attributed to an increased likelihood of hole trapping at the Absorber/HTL interface due to defects^23,27,43^. These findings strongly emphasize the necessity of maintaining a defect density below 10^12^ cm^−3^ at the Absorber/HTL interface to achieve high solar cell efficiency.

Currently, there are no experimental reports on the fabrication of solar cells using the materials CaZrSe_3_, BaZrSe_3_, CaHfSe_3_, and BaHfSe_3_. The only documented solar cell made from chalcogenide perovskite is based on the BaZrS_3_ absorber, which has recently achieved a reported PCE of just 0.17%. However, critical issues such as interface defects in the fabricated solar cell and methods to control these defects have not been investigated. This indicates a significant gap in the existing literature regarding effective strategies to mitigate interface defects specific to chalcogenide perovskite solar cells. Given this situation, it may be helpful to explore techniques from other types of solar cells that could be adapted to address interface defects in these chalcogenide perovskites. Methods like interface engineering and additive engineering are commonly used during solar cell fabrication to minimize recombination losses and defects at ETL/Absorber and HTL/Absorber interfaces. For example, a study by Wang et al. demonstrated that etching Sb_2_(S, Se_3_) with a potassium fluoride solution before depositing the HTL could significantly reduce defects at the HTL/Absorber interface, thereby improving charge carrier transportation^61^. Similarly, research by Hwang et al. highlighted that post-heat treatment of the Cu_2_ZnSn(S, Se)_4 _absorber led to a reduction in interfacial recombination at the ETL/absorber interface, ultimately enhancing the solar cell’s PCE^62^. Moreover, Tian et al. investigated the insertion of interfacial layers on either side of the CsPbI_2_Br perovskite absorber, which was found to passivate defects at both the ETL/Absorber and HTL/Absorber interfaces^63^. In a related study, Yanping and colleagues experimented with doping the perovskite absorber with bromine (Br) to further mitigate interface defects^64^. Overall, drawing from these insights and techniques employed in the fabrication of various types of solar cells, it is plausible that similar methods could effectively address the interface defect issues in solar cells based on CaZrSe_3_, BaZrSe_3_, CaHfSe_3_, and BaHfSe_3_. However, confirmation of suitable techniques for these specific solar cells can only be established following their actual fabrication and subsequent experimental evaluation.

*Comparison of all absorbers*.

**Fig. 14:**
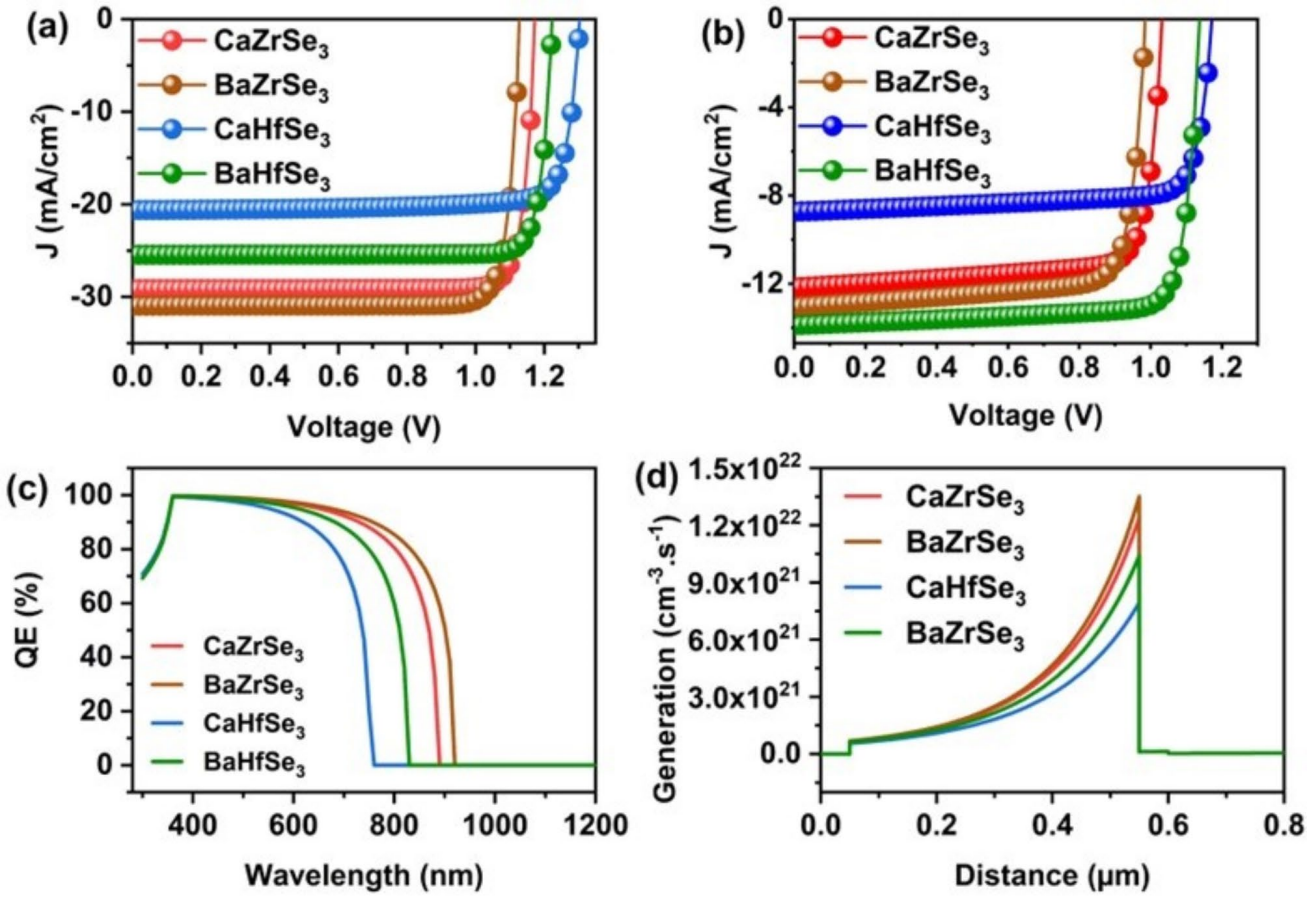
Comparison of J-V characteristics (**a**) Initial, (**b**) Final, (**c**) QE, and (**d**) Recombination rate for novel chalcogenide perovskite solar cells.

This study investigates the solar cell performance of novel chalcogenide perovskites: CaZrSe_3_, BaZrSe_3_, CaHfSe_3_, and BaHfSe_3_. Notably, we achieved impressive final PCEs of 30.08%, 30.58%, 22.53%, and 27.60% for CaZrSe_3_, BaZrSe_3_, CaHfSe_3_, and BaHfSe_3_-based solar cells, respectively. The increase in PCE is primarily attributed to the enhancement in J_SC_, as shown in Fig. 14(a) and 14(b). Specifically, the J_SC_ rises from 12.17 mA/cm^2^, 13.04 mA/cm^2^, 8.73 mA/cm^2^ and 13.88 mA/cm^2^ to 29.16 mA/cm^2^, 30.93 mA/cm^2^, 20.62 mA/cm^2^ and 25.43 mA/cm^2^ for CaZrSe_3_, BaZrSe_3_, CaHfSe_3_ and BaHfSe_3_-based solar cells, respectively. This underscores the significant role of J_SC_, which largely depends on light absorption and charge carrier generation. Furthermore, the QE measurements and generation rates of the proposed solar cells were extracted from SCAPS-1D, as illustrated in Fig. 14(c) and 14(d). The CaZrSe_3_, BaZrSe_3_, CaHfSe_3_, and BaHfSe_3_ solar cells exhibited QEs of 57.78%, 61.10%, 43.94%, and 51.57%, respectively, with the highest generation rates of 1.22 × 10^22^ cm^−3^s^−1^, 1.35 × 10^22^ cm^−3^s^−1^, 7.77 × 10^21^ cm^−3^s^−1^ and 1.02 × 10^22^ cm^−3^s^−1^ at the Absorber/ETL interface (0.54 μm). Notably, BaZrSe_3_ solar cells achieved a high QE and generation rate, which can be attributed to their lower bandgap compared to the other materials, leading to superior performance. Overall, this work demonstrates that all solar cells achieved PCEs greater than 20%, with BaZrSe_3_ and CaZrSe_3_ exceeding 30%. Our findings are likely to inspire material scientists to explore the fabrication of novel CaZrSe_3_, BaZrSe_3_, CaHfSe_3_, and BaHfSe_3_ solar cells.

### Impact of series, shunt resistance, and working temperature for CaZrSe_3_, BaZrSe_3_, CaHfSe_3_, and BaHfSe_3_solar cells

**Fig. 15:**
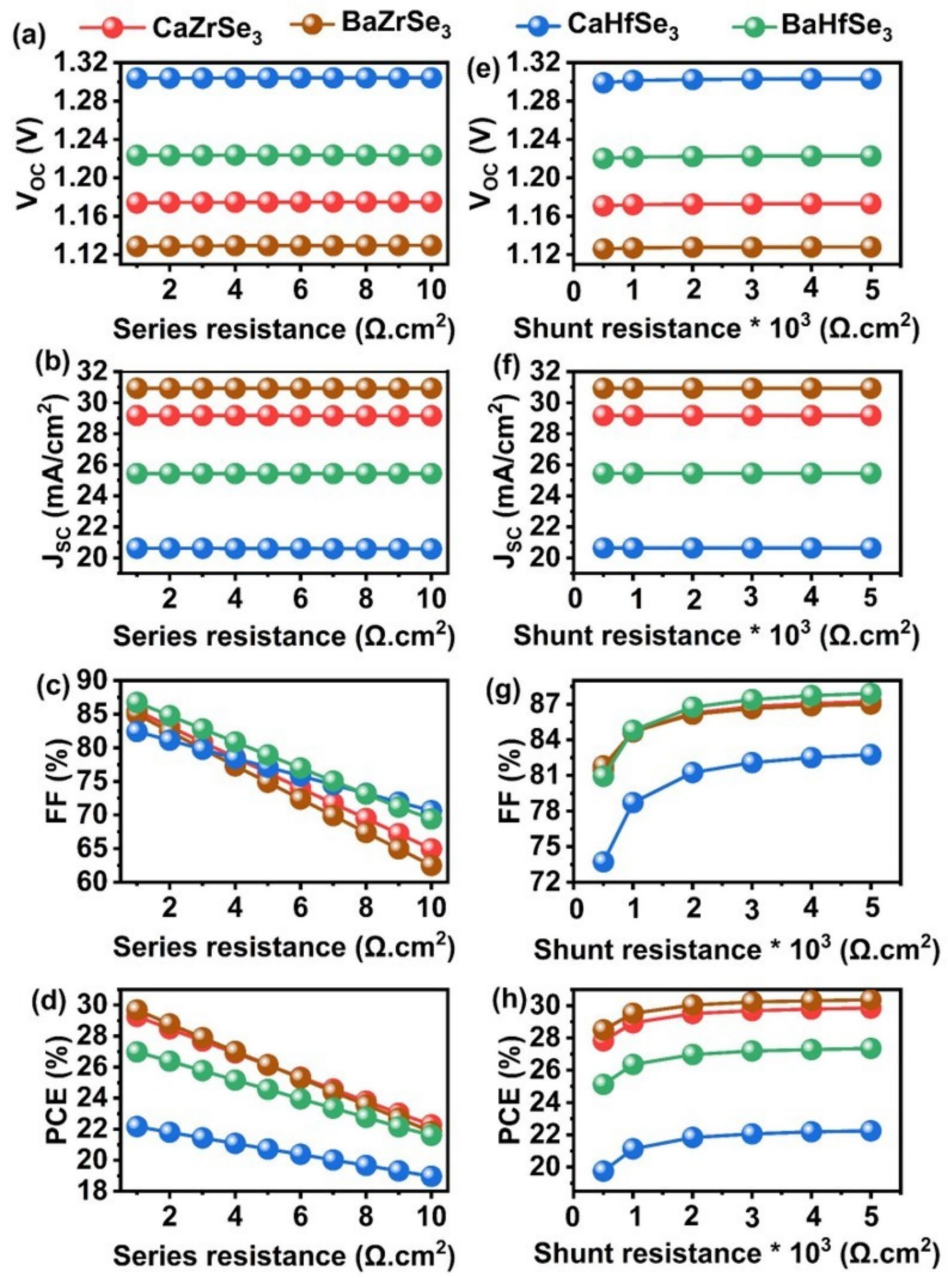
Influence of V_OC_, J_SC_, FF, and PCE as a function of (**a-d**) series resistance and (**e-h**) shunt resistance.

The performance of solar cells is significantly affected by two key factors R_S_ and R_SH_. R_S_ refers to the resistance encountered at metal contacts, transport layers, and the absorber’s resistance outside the space charge region. An increase in R_S_ can lead to a reduction in the overall PCE of solar cells, as it causes some of the current to dissipate as heat instead of being converted into usable electrical energy. To investigate the impact of R_S_, we examined solar cells based on CaZrSe_3_, BaZrSe_3_, CaHfSe_3_, and BaHfSe_3_. We varied R_S_ from 1 to 10 Ω cm², as illustrated in Fig. 15(a-d). It was observed that the V_OC_ and J_SC_ remained largely unchanged across this range. However, the FF decreased significantly, dropping from 85.51%, 85.00%, 82.43%, and 86.74% for CaZrSe_3_, BaZrSe_3_, CaHfSe_3_, and BaHfSe_3_-based solar cells, respectively, to 64.98%, 62.52%, 70.63%, and 69.41%. This decline is attributed to substantial power loss (P_loss_) within the solar cells at higher R_S_ values, as shown in Eq. (7**)**^59,60^ .

7 $$\:{P}_{loss}={I}_{sc}^{2}{R}_{S}$$

The equation above illustrates that P_loss_ is directly proportional to R_S_. This means that an increase in R_S_ results in a higher P_loss_, which in turn reduces the PCE from 29.28 to 22.97%, 29.08 to 21.85%, 22.17 to 18.96%, and 26.99 to 21.60% in the solar cells based on CaZrSe_3_, BaZrSe_3_, CaHfSe_3_ and BaHfSe_3_, respectively. Therefore, an optimal R_S_ of 1 Ω cm² is recommended for the efficient operation of solar cells. On the other hand, the R_SH_ primarily results from factors such as interface barriers, charge-collecting interlayers, metal-based electrodes, and defects or impurities. These issues lead to reverse saturation current in solar cells. Additionally, leakage pathways, like pinholes in the absorber and recombination losses, contribute to R_SH_. The Shockley equation, as represented in Eq. (8**)** and Eq. (9**)**, describes the expected behavior of the J-V characteristics of a solar cell under ideal one-sun illumination conditions^65,66^.

8 $$\:\left[\text{exp}\left(\frac{{q}_{e\:\left(V-\:J{R}_{S}\right)}}{nk{T}_{e}}\right)-1\right]-\:\frac{V-J{R}_{S}}{{R}_{Sh}}$$

9 $$\:\left(\frac{nk{T}_{e}}{{q}_{e}}\right)\text{ln}\left\{\frac{{J}_{PH}}{{J}_{0}}\left(1-\:\frac{{V}_{OC}}{{{R}_{Sh}J}_{PH\:}}\right)\right\}$$

Where q_e_, J_PH_, J_0_, R_S_, R_SH_, n, k, T_e_represent the elementary charge, photocurrent density, density of the reverse bias saturation current, series resistance, shunt resistance, diode ideality factor, Boltzmann constant and ambient temperature, respectively^67^. Thus, R_SH_is altered from 500 to 5000 Ω cm^2^ to investigate its impact on the performance of solar cells. From Fig. 15**(**e-h), it can be noticed that J_SC_ and V_OC_ remain constant while FF and PCE enhance with an increase in R_SH_. In particular, the FF and PCE rise around ~ 7.5% and 2.3%, respectively, for all the solar cells. Therefore, elevated R_SH_ values result in improved solar cell performance.

**Fig. 16:**
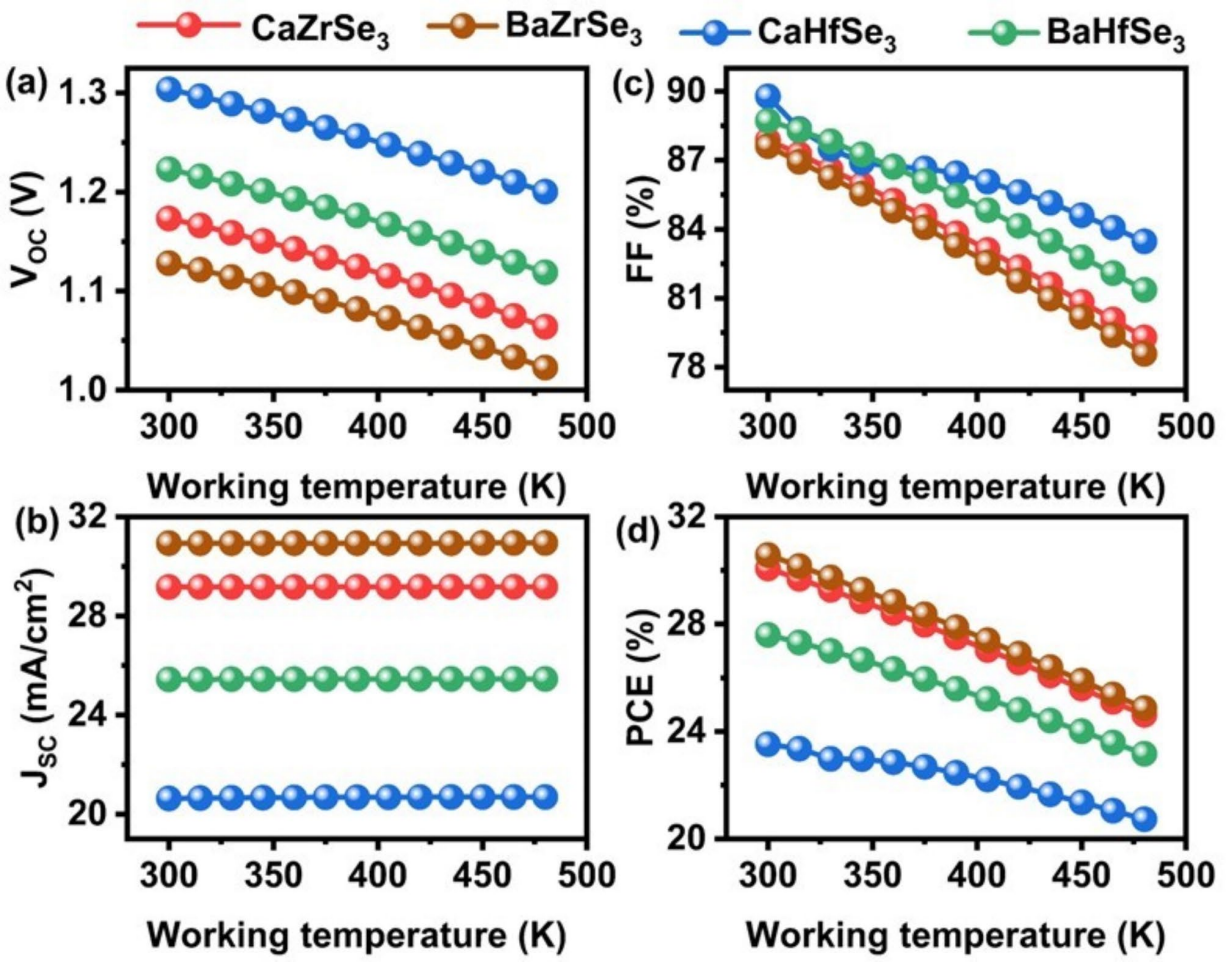
Influence of V_OC_, J_SC_, FF, and PCE as a function of working temperature.

The performance of solar cells often shows instability due to layer deformation at high temperatures^68^. To investigate the relationship between temperature and PV parameters, we varied the temperature from 300 K to 480 K, as shown in Fig. 16 (a-d). The results indicate that as the temperature increases, the V_OC_, FF, and PCE decrease, while the J_SC_ remains relatively constant. The decline in V_OC_ is attributed to the increased vibration of thermally generated electrons at higher temperatures. This heightened vibration makes the electrons less stable and more prone to recombination with holes, leading to an increase in the reverse saturation current (J_0_)^69^. This inverse relationship between V_OC_ and J_0_ is clearly illustrated in Eqs. (10**)**.

10 $$\:{V}_{OC}=\:\frac{nKT}{q}\left(ln\left(1+\:\frac{{J}_{SC}}{{J}_{o}}\right)\right)$$

Where $\:\frac{KT}{q}$ signifies the thermal voltage. Increasing the temperature negatively impacts several physical parameters, including carrier concentration, absorber bandgap, and the mobility of charge carriers. These changes directly affect the efficiency of charge carrier transport, ultimately leading to a reduction in the FF. As a result, the decline in V_OC_ and FF causes the PCE to drop significantly. For instance, PCE decreases from 30.08 to 2.62%, from 30.58 to 24.87%, from 22.53 to 21.36%, and from 27.60 to 23.17% in solar cells based on CaZrSe_3_, BaZrSe_3_, CaHfSe_3_ and BaHfSe_3_, respectively.

## Comparison of SCAPS-1D outcomes with previous studies in the literature

Table 4 presents the outcomes of various theoretical studies on chalcogenide perovskite solar cells using SCAPS-1D. Notably, extensive research has been conducted on BaZrS_3_ chalcogenide perovskite, achieving a maximum PCE of 28.17%. However, there is a need for further exploration of other potential chalcogenide perovskites, highlighting a significant gap in research. Interestingly, we have successfully developed the first-ever CaZrSe_3_, BaZrSe_3_, CaHfSe_3_, and BaHfSe_3_ solar cells by employing TiO_2_, NiO, and au as the ETL, HTL, and metal contact, respectively. Remarkably, these new absorbers yielded unprecedented PCEs of 30.08% for CaZrSe_3_, 30.58% for BaZrSe_3_, 22.74% for CaHfSe_3_, and 27.60% for BaHfSe_3_. This underscores the potential of these emerging absorbers, comparable to that of BaZrS_3_. Thus, our findings are expected to generate interest within the scientific community and contribute to a deeper understanding of the proposed chalcogenide perovskites, providing a framework for developing highly efficient chalcogenide perovskite solar cells.

**Table 4:** Comparison between findings of novel chalcogenide perovskite solar cells with previous studies in the literature

Device structure	V_OC_ (V)	J_SC_ (mA/cm^2^)	FF (%)	PCE (%)	Ref
FTO/TiO_2_/BaZrS_3_/Spiro-OMeTAD/Au	1.21	16.54	86.26	17.29	^70^
FTO/TiO_2_ /BaZrS_3_/Cu_2_O/Au	1.16	12.24	87.13	12.42	^44^
FTO/TiO_2_/BaZrS_3_/Spiro-OMeTAD/Au	0.70	22.00	79.40	12.12	^71^
AZO/i-ZnO/CdS/ BaZrS_3_/a-Si	1.31	19.08	78.88	19.72	^72^
FTO/TiO_2_/BaZrSe_3_/Spiro-OMeTAD/Au	0.72	46.65	77.32	25.84	^71^
FTO/TiO_2_/BaZrS_3_/Spiro-OMeTAD/Au	1.08	16.80	88.60	16.07	^73^
FTO/TiO_2_/BaZrSe_3_/Spiro-OMeTAD/Au	0.69	33.05	81.39	19.76	^16^
FTO/ZrS_2_/BaZrS_3_/SnS/Pt	1.18	29.74	80.15	28.17	^23^
FTO/ZnO /SrZrS_3_/NiO/Ni	1.18	26.13	84.29	25.97	^47^
FTO/ SnO_2_/SrZrS_3_/Cu-MOF/Ni	1.17	29.54	88.40	30.60	^27^
FTO/ TiO_2_/CaZrSe_3_/NiO/Au	1.17	29.16	87.88	30.08	*
FTO/ TiO_2_/BaZrSe_3_/NiO/Au	1.12	30.93	87.61	30.58	*
FTO/ TiO_2_/CaHfSe_3_/NiO/Au	1.30	20.62	83.78	22.53	*
FTO/ TiO_2_/BaHfSe_3_/NiO/Au	1.22	25.43	88.72	27.60	*
The * represents the findings of the present work					

## Conclusion

This study systematically investigated novel CP solar cells using CaZrSe_3_, BaZrSe_3_, CaHfSe_3_ and BaHfSe_3_ absorbers via SCAPS-1D. We began by evaluating the performance of these solar cells by varying the thickness, carrier concentration, and defect density of the ETL, absorbers, and HTL. By adjusting the carrier concentration in the ETL, we created deep energy levels at the interfaces, which reduced non-radiative recombination and enhanced PCE to 11.73%, 11.99%, 10.03%, and 14.49% for CaZrSe_3_, BaZrSe_3_, CaHfSe_3_ and BaHfSe_3_ solar cells, respectively. Optimizing the absorber’s thickness and carrier concentration increased light absorption by approximately 11.93% and improved conductivity and built-in potential, facilitating charge carrier transfer. We achieved a significant PCE increase of about 5.1% for all solar cells at an optimized HTL thickness of 50 nm, a carrier concentration of 10^20^ cm^−3^, and a defect density of 10^16^ cm^−3^. This enhancement was due to the increased electric field at the Absorber/HTL interface, which effectively reduced the barrier for charge carriers. After fine-tuning the thickness, carrier concentration, and defect density of all layers, we achieved maximum PCEs of 30.08%, 30.58%, 22.74%, and 27.60% for CaZrSe_3_, BaZrSe_3_, CaHfSe_3_ and BaHfSe_3_, respectively. Both CaZrSe_3_ and BaZrSe_3_-based solar cells surpassed a PCE of 30%, highlighting their superiority over other solar cells due to their narrow bandgap, which leads to improved light absorption and a higher generation rate of charge carriers, resulting in elevated J_SC_ of approximately 29 mA/cm^2^. Overall, we anticipate that our research will generate significant interest among material scientists globally regarding the advancement of novel CaZrSe_3_, BaZrSe_3_, CaHfSe_3_ and BaHfSe_3_ CP solar cells.

## Data Availability

Required data are available from the corresponding author upon reasonable request.

## References

[1] Zhang, J., Gao, X., Deng, Y., Zha, Y. & Yuan, C. Comparison of life cycle environmental impacts of different perovskite solar cell systems. *Sol Energy Mater. Sol Cells*. **166**, 9–17 (2017).

[2] Renewable, E. & Laboratory, N. *NREL Best Research-Cell PV Efficiency Chart*. (1976).

[3] Wang, X., Zhang, T., Lou, Y. & Zhao, Y. All-inorganic lead-free perovskites for optoelectronic applications. *Mater. Chem. Front.* **3**, 365–375 (2019).

[4] Reza, M. S. et al. Design and optimization of High-Performance Novel RbPbBr_3_-Based solar cells with Wide-Band-Gap S-Chalcogenide Electron Transport Layers (ETLs). *ACS Omega*. **9**, 19824–19836 (2024). 38737037 10.1021/acsomega.3c08285 PMC11079912

[5] Sun, Y. Y., Agiorgousis, M. L., Zhang, P. & Zhang, S. Chalcogenide perovskites for Photovoltaics. *Nano Lett.* **15**, 581–585 (2015). 25548882 10.1021/nl504046x

[6] Han, Y. et al. Preparation of chalcogenide perovskite SrHfS_3_ and luminescent SrHfS_3_:Eu^2+^ thin films. *Appl. Phys. Lett.* **124**, 13 (2024).

[7] Liang, Y. et al. Tapping the light emitting potential of Chalcogenide Perovskite SrHfS_3_ via Eu ^2+^ doping. *Adv. Opt. Mater.* **12**, 2301977 (2024).

[8] Yu, Z. et al. Chalcogenide Perovskite Thin films with controlled phases for Optoelectronics. *Adv. Funct. Mater.* **34**, 2309514 (2024).

[9] Comparotto, C., Ström, P., Donzel-Gargand, O., Kubart, T. & Scragg, J. J. S. Synthesis of BaZrS_3_ Perovskite Thin films at a moderate temperature on Conductive substrates. *ACS Appl. Energy Mater.* **5**, 6335–6343 (2022).

[10] Yu, Z. et al. Chalcogenide Perovskite BaZrS_3_ thin-film electronic and optoelectronic devices by low temperature processing. *Nano Energy*. **85**, 105959 (2021).

[11] Romagnoli, L. et al. A simple synthetic approach to BaZrS_3_, BaHfS_3_, and their solid solutions. *J. Am. Ceram. Soc.* **107**, 698–703 (2024).

[12] Yang, R., Jess, A. D., Fai, C. & Hages, C. J. Low-Temperature, solution-based synthesis of luminescent Chalcogenide Perovskite BaZrS_3_ nanoparticles. *J. Am. Chem. Soc.* **144**, 15928–15931 (2022). 36000912 10.1021/jacs.2c06168

[13] Zilevu, D., Parks, O. O. & Creutz, S. E. Solution-phase synthesis of the chalcogenide perovskite barium zirconium sulfide as colloidal nanomaterials. *Chem. comm.* **58**, 10512–10515 (2022). 36043522 10.1039/d2cc03494h

[14] Vincent, K. C., Agarwal, S., Turnley, J. W. & Agrawal, R. Liquid flux–assisted mechanism for Modest Temperature synthesis of large-grain BaZrS_3_ and BaHfS_3_ Chalcogenide Perovskites. *Adv. Energy Sustain. Res.* **4**, 2300010 (2023).

[15] Dallas, P. et al. Exploring the potential of powder-to-film processing for proof-of-concept BaZrS_3_ perovskite solar cells. *Mater. Today Commun.* **39**, 108608 (2024).

[16] Thakur, N., Kumar, P. & Sharma, P. Simulation study of chalcogenide perovskite (BaZrSe_3_) solar cell by SCAPS-1D. *Mater. Today Proc.* (2023).

[17] Singh, N., Agarwal, A. & Agarwal, M. Numerical simulation of highly efficient lead-free all-perovskite tandem solar cell. *Sol Energy*. **208**, 399–410 (2020).

[18] Banik, S., Das, A., Das, B. K. & Islam, N. Numerical simulation and performance optimization of a lead-free inorganic perovskite solar cell using SCAPS-1D. *Heliyon* 10, e23985 (2024). 10.1016/j.heliyon.2024.e23985 PMC10805918 38268575

[19] Danladi, E. et al. Impact of hole transport material on perovskite solar cells with different metal electrode: a SCAPS-1D simulation insight. *Heliyon* **9**, e16838 (2023). 37313155 10.1016/j.heliyon.2023.e16838 PMC10258428

[20] Kumari, R., Mamta, M., Kumar, R., Singh, Y. & Singh, V. N. 24% efficient, simple ZnSe/Sb_2_Se_3_ Heterojunction Solar cell: an analysis of PV characteristics and defects. *ACS Omega*. **8**, 1632–1642 (2023). 36643481 10.1021/acsomega.2c07211 PMC9835802

[21] Arockiya-Dass, K. T., Sekar, K. & Marasamy, L. Theoretical insights of degenerate ZrS_2_ as a new buffer for highly efficient Emerging Thin-Film Solar cells. *Energy Technol.* **11**, (2023).

[22] Rahman, M. F. et al. Improving the efficiency of a CIGS solar cell to above 31% with Sb_2_S_3_ as a new BSF: a numerical simulation approach by SCAPS-1D. *RSC Adv.* **14**, 1924–1938 (2024). 38192318 10.1039/d3ra07893k PMC10772862

[23] Vincent Mercy, E. N., Srinivasan, D. & Marasamy, L. Emerging BaZrS_3_ and ba(zr,Ti)S_3_ Chalcogenide Perovskite Solar cells: a Numerical Approach toward device Engineering and Unlocking Efficiency. *ACS Omega*. **9**, 4359–4376 (2024). 38313502 10.1021/acsomega.3c06627 PMC10832013

[24] Sekar, K., Marasamy, L., Mayarambakam, S., Selvarajan, P. & Bouclé, J. Highly efficient lead-free silver bismuth iodide (Ag_3_BiI_6_) rudorffite solar cells with novel device architecture: a numerical study. *Mater. Today Commun.* **38**, 108347 (2024).

[25] Hossain, M. K. et al. Achieving above 24% efficiency with non-toxic CsSnI _3_ perovskite solar cells by harnessing the potential of the absorber and charge transport layers. *RSC Adv.* **13**, 23514–23537 (2023). 37546214 10.1039/d3ra02910g PMC10402874

[26] Arockiya Dass, K. T., Hossain, M. K. & Marasamy, L. Highly efficient emerging Ag_2_BaTiSe_4_ solar cells using a new class of alkaline earth metal-based chalcogenide buffers alternative to CdS. *Sci. Rep.* **14**, 1473 (2024). 38233504 10.1038/s41598-024-51711-6 PMC10794422

[27] Linda, E., Rasu Chettiar, A. D. & Marasamy, L. Emerging class of SrZrS_3_ chalcogenide perovskite solar cells: conductive MOFs as HTLs - A game changer? *Sol Energy Mater. Sol Cells*. **278**, 113204 (2024).

[28] Osei-Agyemang, E., Enninful Adu, C. & Balasubramanian, G. Doping and Anisotropy–Dependent Electronic Transport in Chalcogenide Perovskite CaZrSe_3_ for high thermoelectric efficiency. *Adv. Theory Simul.* **2**, 1900060 (2019).

[29] Odeh, Y. M. et al. Tuning the bandgap of cubic and orthorhombic BaZrS_3_ by substituting sulfur with selenium. *AIP Adv.* **13**, (2023).

[30] Rahman, M. Design and simulation of a high-performance Cd-free Cu_2_SnSe_3_ solar cells with SnS electron-blocking hole transport layer and TiO_2_ electron transport layer by SCAPS-1D. *SN Appl. Sci.* **3**, 253 (2021).

[31] Mouchou, R. T., Jen, T. C., Laseinde, O. T. & Ukoba, K. O. Numerical simulation and optimization of p-NiO/n-TiO_2_ solar cell system using SCAPS. *Mater. Today Proc.* **38**, 835–841 (2021).

[32] Mandadapu, U. Performance analysis of NiO-based perovskite solar cell model. *Mater. Today Proc.* **25**, (2023).

[33] Islam, S. Defect study and modelling of SnX_3_-Based Perovskite Solar cells with SCAPS-1D. *Nano Mater.* **11**, 1218 (2021). 10.3390/nano11051218 PMC8147994 34063020

[34] Ukoba, K. O., Eloka-Eboka, A. C. & Inambao, F. L. Review of nanostructured NiO thin film deposition using the spray pyrolysis technique. *Renew. Sustain. Ener*. **82**, 2900–2915 (2018).

[35] Zhang, K. H. L. et al. Electronic structure and Band Alignment at the NiO and SrTiO_3_ p–n heterojunctions. *ACS Appl. Mater. Interfaces*. **9**, 26549–26555 (2017). 28695740 10.1021/acsami.7b06025

[36] Chen, Q., Ni, Y., Dou, X. & Yoshinori, Y. The Effect of Energy Level of Transport Layer on the performance of Ambient Air Prepared Perovskite Solar cell: a SCAPS-1D Simulation Study. *Cryst. (Basel)*. **12**, 68 (2022).

[37] El-Naggar, A. A. et al. Numerical simulation-based performance enhancement approach for an inorganic BaZrS_3_/CuO heterojunction solar cell. *Sci. Rep.* **14**, 7614 (2024). 38556524 10.1038/s41598-024-57636-4 PMC10982297

[38] Tavakoli, M. M., Yadav, P., Tavakoli, R. & Kong, J. Surface Engineering of TiO _2_ ETL for highly efficient and Hysteresis-less Planar Perovskite Solar Cell (21.4%) with enhanced Open‐Circuit Voltage and Stability. *Adv. Energy Mater.*, **8**, (2018).

[39] Rana, M. S., Islam, M. M. & Julkarnain, M. Enhancement in efficiency of CZTS solar cell by using CZTSe BSF layer. *Sol. Energy*. **226**, 272–287 (2021).

[40] Qasim Agha, D. N. & Algwari, Q. T. The influence of the interface layer between the electron transport layer and absorber on the performance of perovskite solar cells. *IOP Conf. Ser. Mater. Sci. Eng.* **1152**, 012033 (2021).

[41] Córdoba, M. & Taretto, K. Insight into the dependence of Photovoltaic Performance on Interfacial Energy Alignment in Solar cells with Mobile ions. *Sol RRL*. **8**, 2300742 (2024).

[42] Saadat, M., Amiri, O. & Mahmood, P. H. Potential efficiency improvement of CuSb(S1-x,S)_2_ thin film solar cells by the zn(O,S) buffer layer optimization. *Sol Energy*. **225**, 875–881 (2021).

[43] Linda, E., Rasu Chettiar, A. D. & Marasamy, L. Theoretical insights into high-efficiency BaZr_0.96_Ti_0.04_S_3_ chalcogenide perovskite solar cells using phthalocyanine HTLs. *Mater. Lett.* **375**, 137203 (2024).

[44] Karthick, S., Velumani, S. & Bouclé, J. Chalcogenide BaZrS_3_ perovskite solar cells: a numerical simulation and analysis using SCAPS-1D. *Opt. Mater. (Amst)*. **126**, 112250 (2022).

[45] Du, H. J., Wang, W. C. & Zhu, J. Z. Device simulation of lead-free CH_3_NH_3_SnI_3_ perovskite solar cells with high efficiency. *Chin. Phys. B*. **25**, 108802 (2016).

[46] Abdelaziz, S., Zekry, A., Shaker, A. & Abouelatta, M. Investigating the performance of formamidinium tin-based perovskite solar cell by SCAPS device simulation. *Opt. Mater. (Amst)*. **101**, 109738 (2020).

[47] Chawki, N., Rouchdi, M., Alla, M. & Fares, B. Simulation and analysis of high-performance hole transport material SrZrS_3_-based perovskite solar cells with a theoretical efficiency approaching 26%. *Sol Energy*. **262**, 111913 (2023).

[48] Nishigaki, Y. et al. Extraordinary strong Band-Edge absorption in distorted Chalcogenide Perovskites. *Solar RRL*. **4**, 1852362 (2020).

[49] Dixit, H., Bansal, N. K., Porwal, S., Kumar, D. & Singh, T. Performance assessment of earth-abundant Kesterite and lead-free chalcogenide-based perovskite solar cells using SCAPS-1D. *Optik (Stuttg)*. **295**, 171474 (2023).

[50] Swarnkar, A. et al. Are Chalcogenide Perovskites an Emerging Class of Semiconductors for Optoelectronic properties and Solar Cell? *Chem. Mater.* **31**, 565–575 (2019).

[51] Duan, H. et al. The role of Sulfur in Solution-processed Cu_2_ZnSn(S,Se)_4_ and its effect on defect Properties. *Adv. Funct. Mater.* **23**, 1466–1471 (2013).

[52] Haider, S. Z., Anwar, H. & Wang, M. Theoretical device Engineering for High-Performance Perovskite Solar cells using CuSCN as Hole Transport Material Boost the Efficiency above 25%. *Physi Status Solidi (a)*. **216**, 1900102 (2019).

[53] Meng, W. et al. Alloying and defect control within Chalcogenide perovskites for optimized photovoltaic application. *Chem. Mater.* **28**, 821–829 (2016).

[54] Shasti, M. & Mortezaali, A. Numerical Study of Cu_2_O, SrCu_2_O_2_, and CuAlO_2_ as hole-transport materials for application in Perovskite Solar cells. *Physi Status Solidi (a)*. **216**, 81093 (2019).

[55] Sharma, A. K. & Kaushik, D. K. Numerical simulation of MASnI_3_ /CuI heterojunction based perovskite solar cell. *J. Phys. Conf. Ser.* **2267**, 012148 (2022).

[56] Yuan, Y. et al. Shallow defects and variable photoluminescence decay times up to 280 µs in triple-cation perovskites. *Nat. Mater.* **23**, 391–397 (2024). 38195863 10.1038/s41563-023-01771-2 PMC10917677

[57] Saadat, M. & Amiri, O. Fine adjusting of charge carriers transport in absorber/HTL interface in Sb_2_(S,Se)_3_ solar cells. *Sol Energy*. **243**, 163–173 (2022).

[58] Baiano, C. et al. Role of surface defects in CO_2_ adsorption and activation on CuFeO_2_ delafossite oxide. *Mol. Catal.* **496**, 111181 (2020).

[59] Khan, T. M., Ahmed, S. R. & Al Investigating the performance of FASnI _3_ -Based Perovskite Solar cells with Various Electron and hole transport layers: Machine Learning Approach and SCAPS‐1D analysis. *Adv. Theory Simul.* **190**, 2400353 (2024).

[60] Hosen, A., Mian, M. S., Ahmed, S. R. & Al Improving the performance of lead-free FASnI _3_ ‐Based Perovskite Solar cell with nb _2_ O _5_ as an Electron Transport Layer. *Adv. Theory Simul.* **6**, 853612 (2023).

[61] Wang, X. et al. Chemical etching induced surface modification and gentle gradient bandgap for highly efficient Sb_2_(S,Se)_3_ solar cell. *Appl. Surf. Sci.* **579**, 152193 (2022).

[62] Hwang, S. K. et al. Improved interfacial properties of electrodeposited Cu_2_ZnSn(S,Se)_4_ thin-film solar cells by a facile post‐heat treatment process. *Prog Photovolt: Res. App*. **28**, 1345–1354 (2020).

[63] Jayan, K. High-efficiency non‐toxic 2‐Terminal and 4‐Terminal Perovskite‐Transition Metal Dichalcogenide Tandem Solar cells. *Adv. Theory Simul.* **5**, 122350 (2022).

[64] Raj, A., Kumar, M., Kumar, A., Laref, A. & Anshul, A. Investigating the potential of lead-free double perovskite Cs_2_AgBiBr_6_ material for solar cell applications: a theoretical study. *Int. J. Energy Res.* **46**, 13801–13819 (2022).

[65] Chawki, N., Rouchdi, M. & Fares, B. Numerical Study of BaZrS_3_ based Chalcogenide Perovskite Solar Cell using SCAPS-1D device Simulation. *Res. Square*. **183**, 833140 (2022).

[66] Karthick, S., Velumani, S. & Bouclé, J. Chalcogenide BaZrS3 perovskite solar cells: a numerical simulation and analysis using SCAPS-1D. *Opt. Mater. (Amst)*. **126**, 112250 (2022).

[67] Goutham Kumar, S., Pramod, A. & Honnavar, G. V. R, P. C. Proposal for a novel perovskite solar cell based on BaZrS3 with optimized electron and hole transport layer using SCAPS-1D. *Chem. Bull.* 10289–10307. (2023).

[68] Thakur, N. et al. Recent advances in BaZrS3 perovskites: synthesis, properties, and future trends. *J. Alloys Compd.* **957**, 170457 (2023).

[69] Barman, B. & Ingole, S. Analysis of Si back-contact for Chalcogenide Perovskite Solar cells based on BaZrS_3_ using SCAPS‐1D. *Adv. Theory Simul.* **6**, (2023).

[70] Chawki, N. et al. Efficacy analysis of BaZrS_3_ -based perovskite solar cells: investigated through a numerical simulation. *Adv. Mater. Process. Technol.* **83**, 1–14 (2024).

[71] Thakur, N., Kumar, P. & Sharma, P. Simulation study of chalcogenide perovskite (BaZrSe_3_) solar cell by SCAPS-1D. *Mater. Today Proc.* **62**, 93487 (2023).

